# Aberrant ER-mitochondria communication is a common pathomechanism in mitochondrial disease

**DOI:** 10.1038/s41419-024-06781-9

**Published:** 2024-06-10

**Authors:** Patricia Morcillo, Khushbu Kabra, Kevin Velasco, Hector Cordero, Sarah Jennings, Taekyung D. Yun, Delfina Larrea, H. Orhan Akman, Eric A. Schon

**Affiliations:** 1 Department of Neurology, Columbia University Medical Center, New York, NY 10032 USA; 2 Columbia Center for Translational Immunology, Columbia University Medical Center, New York, NY 10032 USA; 3 Immunology Group, Department of Physiology, Faculty of Veterinary, University of Extremadura, Caceres, 10003 Spain; 4 Stony Brook University, Stony Brook, New York, NY 11794 USA; 5 Department of Genetics and Development, Columbia University Medical Center, New York, NY 10032 USA

**Keywords:** Diseases, Neurological disorders

## Abstract

Genetic mutations causing primary mitochondrial disease (i.e those compromising oxidative phosphorylation [OxPhos]) resulting in reduced bioenergetic output display great variability in their clinical features, but the reason for this is unknown. We hypothesized that disruption of the communication between endoplasmic reticulum (ER) and mitochondria at mitochondria-associated ER membranes (MAM) might play a role in this variability. To test this, we assayed MAM function and ER-mitochondrial communication in OxPhos-deficient cells, including cybrids from patients with selected pathogenic mtDNA mutations. Our results show that each of the various mutations studied indeed altered MAM functions, but notably, each disorder presented with a different MAM “signature”. We also found that mitochondrial membrane potential is a key driver of ER-mitochondrial connectivity. Moreover, our findings demonstrate that disruption in ER-mitochondrial communication has consequences for cell survivability that go well beyond that of reduced ATP output. The findings of a “MAM-OxPhos” axis, the role of mitochondrial membrane potential in controlling this process, and the contribution of MAM dysfunction to cell death, reveal a new relationship between mitochondria and the rest of the cell, as well as providing new insights into the diagnosis and treatment of these devastating disorders.

## Introduction

Mitochondrial diseases encompass a diverse range of disorders caused by mutations in either nuclear DNA (nDNA) or mitochondrial DNA (mtDNA), both of which encode mitochondrial proteins. Unlike nDNA, mtDNA is maternally-inherited and is present in multiple copies per cell [1]. Mitochondrial diseases due to mutations in mtDNA include point mutations and DNA rearrangements (such as deletions, duplications, or inversions) that directly compromise the function of the oxidative phosphorylation (OxPhos) system. Epidemiological studies of pathogenic mtDNA mutations in adults have shown a minimum prevalence of approximately 1 in 5000, making mitochondrial diseases among the most common genetic disorders [2]. Deleterious heteroplasmic mtDNA mutations (in which the patient carries both normal and mutated mitochondrial DNAs in the same cell or tissue) cause a biochemical phenotype only if present in high proportions. For unknown reasons, this “threshold effect” varies among mutation types; for example, in skeletal muscle the mutation load for a mutation in a tRNA (~90%) [3, 4] is typically higher than that for a large-scale partial deletion of mtDNA (~55–85%) [5, 6]. Currently, there is no effective therapy for most mitochondrial diseases, many of which are ultimately fatal [7], and most treatments are only palliative [8].

The endoplasmic reticulum (ER) communicates with other compartments of the cell, including mitochondria [9]. This communication occurs in a specialized sub-compartment of the ER called mitochondria-associated ER membranes (MAM), which is an intracellular lipid raft-like domain that regulates cellular cholesterol, phospholipid, and calcium homeostasis, and mitochondrial bioenergetics [10–12]. Interestingly, altered ER-mitochondrial communication is associated with impaired mitochondrial respiration in a number of pathologies, including neurogenerative diseases [13–16]. Equally important, respiratory chain (R.C.) and OxPhos deficits in these disorders are associated with both increased and decreased ER-mitochondrial connectivity [17–19]. For example, in cell models of Alzheimer disease (AD), reduced respiration was associated with the upregulation of MAM-resident metabolic enzymes as a consequence of increased ER-mitochondrial apposition [13, 17]. These data indicate that oxidative energy metabolism is dynamic, as it responds to the modulation of communication between mitochondria and ER.

While we know that altered MAM function can affect bioenergetic output, there is no information on the opposite possibility, namely, whether perturbed bioenergetics can affect MAM function, although one report suggests that a pathogenic mutation in mtDNA affects ER-mitochondrial connectivity [20]. To address this gap, we assayed MAM function in a variety of homoplasmic cybrid cell lines repopulated with different patient-derived pathogenic mutant mtDNAs, as well as in fibroblasts obtained from a mitochondrial disease patient with a pathogenic nDNA mutation. For this, we measured two aspects of MAM function, namely, synthesis/transport of phospholipids and the conversion of cholesterol to cholesteryl esters. Notably, we found that MAM function was perturbed significantly in each disorder studied, although each had a different MAM “signature”. We also provide evidence that mitochondrial membrane potential plays a key role in the regulation of ER-mitochondrial connectivity. In addition, our findings show that alterations in ER-mitochondrial communication have a negative impact on cell survival. Finally, our findings point to the possibility of targeting ER-mitochondrial contacts as a mode of therapy for mitochondrial diseases.

## Materials and methods

### Cell lines and cultures

A cell line lacking mtDNA (ρ^0^; denoted 143B206) derived from the human osteosarcoma cell line containing mtDNA (ρ^+^; denoted 143B) [21] was fused with enucleated cells from patients diagnosed with common mtDNA-related disorders, to produce cytoplasmic hybrid (cybrid) lines, as described previously [22]. Homoplasmic wild-type (WT, ~0% mutation) and homoplasmic mutant (~100% mutation) cybrid clones were obtained from a patient with Kearns-Sayre syndrome with a partial deletion of mtDNA [Δ-mtDNA] that removes 1902 bp of the mtDNA; denoted FLP6a39.32) [23], and maternally-inherited Leigh syndrome (MILS) with a T8993G point mutation in the ATPase6 gene [24] (denoted JCP261) (see Table in Supplementary Fig. 1A). To confirm homoplasmy (or more precisely, the absence of heteroplasmy, accurate to ~99%), we measured the content of mtDNA by quantitative PCR (qPCR), by DNA sequencing, and by failure to grow in medium lacking uridine and pyruvate [21]. To confirm homoplasmy, these assays were also routinely performed during the course of the experiments. We also confirmed the functional consequence of the various homoplasmic mutations by showing that they had severe reductions in oxygen consumption rate (OCR) vs the corresponding WT cells, using the Seahorse assay (Supplementary Fig. 1B). The cybrid cells were grown in Dulbecco’s Modified Eagle’s Medium (DMEM) containing 10% fetal bovine serum (FBS) + 50 U/ml penicillin/streptomycin and 50 μg/ml uridine and pyruvate at 37 °C with 5% CO_2_.

Experiments with primary skin fibroblasts were obtained with verbal informed consent from two healthy 3-year-old females and a 35-year-old female (as controls), and from a 3-year old female patient with a mutation in the nuclear gene *NDUFS4*, affecting a subunit of complex I of the respiratory chain (c.355 G > A (p.Asp119His) [25] (see Table in Supplementary Fig. 1A). Fibroblasts were maintained in DMEM containing 10% FBS + 50 U/ml penicillin/streptomycin at 37 °C with 5% CO_2_. All cells were routinely tested for mycoplasma contamination.

### Phospholipid transfer assay

In order to measure ER-mitochondrial communication at the MAM, we incubated cells for 2 h in serum-free medium to ensure removal of exogenous lipids and serine. The medium was then replaced with MEM containing 2.5 μCi/ml ^3^H-serine (Perkin Elmer NET248005MC). After a period of incubation for 2, 4, and 6 h, cells were washed with PBS. Lipid extraction was done by a modification of the Bligh and Dyer method [26]. Briefly, three volumes of chloroform/methanol (2:1 v/v) were added to the samples and vortexed. After centrifugation at 8000 g for 5 min, the organic phase was washed twice with two volumes of methanol/water (1:1 v/v), and the organic phase was dried under nitrogen (N_2_). Dried lipids were resuspended in 40 μl chloroform/methanol (2:1 v/v) and applied to a thin-layer chromatography (TLC) plate. To analyze PtdSer and PtdEtn, the chromatography was run in two phases composed of petroleum ether/diethyl ether/acetic acid (84:15:1 v/v/v) and chloroform/methanol/acetic acid/water (60:50:1:4 v/v/v/v). Development was performed by exposure of the plate to iodine vapor to visualize the various lipid classes (iodine binds to unsaturated bonds in lipids, so saturated lipids, while present, will not be visualized on the TLC plate). Spots corresponding to PtdSer and PtdEtn (using appropriate lipid standards; Sigma #P7769 and #P60648) were cut carefully from the plate and quantified in a scintillation counter. Data were represented as ^3^H counts per minute per unit of total protein.

### ACAT1 activity assay

A second, complementary, measure of MAM functionality is the synthesis of cholesteryl esters (CE) by acyl-CoA:cholesterol acyltransferase 1 (ACAT1; gene *SOAT1*), a MAM-localized enzyme [27]. To measure ACAT activity, whole cells were pre-incubated for 2 h in serum-free medium to remove all exogenous lipids. After that, the medium was removed and replaced with serum-free DMEM containing 2 μCi/ml of ^3^H-cholesterol (Perkin Elmer NET139001MC) or 2.5 μCi/ml of ^3^H-oleic acid (Perkin Elmer NET289001MC) solubilized in 2% bovine serum albumin (fatty acid free-BSA [FAF-BSA]; Sigma A3803) that was previously allowed to equilibrate for 30 min at 37 °C. The cells were incubated with the radiolabeled medium for 2, 4, and 6 h. After that, cells were washed 3 times in PBS. Lipids were extracted by the Bligh and Dyer method [26] and dried under N_2_. Total lipids were dissolved in 40 μl chloroform/methanol (2:1 v/v) and TLC was performed using free cholesterol (Sigma C8667) and cholesteryl palmitate (Sigma C6072) as migration standards. Plates were developed with hexane:diethylether:acetic acid (80:20:1 v/v/v) and the isolated bands were quantified in a scintillation counter [13]. Data were represented as ^3^H counts per minute per unit of total protein.

### Assay of other lipid species

For analysis of trigylcerides (TGA) and free fatty acids (FFA), cells were treated in medium containing ^3^H-oleic acid, as above, and bands corresponding to these species were isolated and quantitated, as described above.

### Lipid droplet staining

Staining of lipid droplets (LDs) was performed using HCS LipidTox™ Green neutral lipid stain (Invitrogen H34475), which visualizes neutral lipids such as TGA and CE, according to the manufacturer’s instructions. Cells were detached by trypsin/EDTA solution and fixed with 4% paraformaldehyde (PFA; Santa Cruz Biotechnology sc-281692) for 10 min at room temperature (RT). Following fixation, cells were washed three times in PBS and resuspended in 200 μl fresh medium with 1 μM LipidTox, and incubated for 30 min at RT. The samples were then acquired in a flow cytometer (BD Fortessa), and the median green fluorescence intensity for each sample was determined. Data were analyzed using FCS Express 6 Research Edition (DeNovo Software).

For imaging, cells were stained with LipidTox as described above and mounted with Fluoromount-G™ (Thermo Fisher Scientific 00-4958-02). The images were acquired with a Leica SP8 point scanning confocal microscope (40× objective), and several images were automatically stitched together using Leica LASX Software. At least 3 coverslips for each biological replicate per group, and multiple areas per chamber, selected on a random basis, were used for quantification analysis. The fluorescence intensity for a given cell was quantified and normalized to the number of cells, using Image J software.

### Lipidomic analysis

Lipids were extracted from equal amounts of material (30 μg protein/sample). Lipid extracts were prepared via chloroform–methanol extraction, spiked with appropriate internal standards, and analyzed using a 6490 Triple Quadrupole LC/MS system (Agilent Technologies, Santa Clara, CA) as described previously [28]. Cholesterol and cholesteryl esters were separated by normal-phase HPLC using an Agilent Zorbax Rx-Sil column under the following conditions: mobile phase A (chloroform:methanol: 1 M ammonium hydroxide, 89.9:10:0.1, v/v/v) and mobile phase B (chloroform:methanol:water:ammonium hydroxide, 55:39.9:5:0.1, v/v/v/v); 95% A for 2 min, linear gradient to 30% A over 18 min and held for 3 min, and linear gradient to 95% A over 2 min and held for 6 min. Quantification of lipid species was accomplished using multiple reaction monitoring transitions that were developed in earlier studies [28] in conjunction with referencing of appropriate internal standards. Values were represented as mole fraction % with respect to total lipids (i.e., % molarity), where the lipid mass of any specific lipid was normalized to the total mass of all lipids measured [28]. Results were expressed as normalized z-score in the heatmap plot.

### Lipid droplet isolation

All procedures were performed using pre-chilled equipment and solutions [29]. Cells were plated in ten 100-mm dishes, rinsed in cold PBS, scraped, and resuspended in 2 ml hypotonic lysis medium (HLM) with sucrose-HEPES-EGTA buffer. The preparation was then homogenized mechanically with 9 strokes in a glass-teflon Dounce homogenizer. The homogenate was then transferred to a 50-ml Falcon tube and centrifuged at 1000 g for 10 min at 4 °C. The supernatant was carefully poured into a new ice-cold Falcon tube and resuspended in HLM containing 60% sucrose. The suspension was layered on a sucrose gradient in a 13.2-ml ultracentrifuge tube placed in an SW41Ti rotor and centrifuged at 28,000 g for 30 min at 4 °C. The pellets were re-suspended in HLM and the protein concentration was determined by Bradford, as described below.

### Plasmids and transfections

The mammalian expression plasmid MAMtracker-Green with an appended C-terminal HA epitope tag (pMAMtracker-Green) [30] was kindly provided by Dr. Koji Yamanaka (Nagoya University). Briefly, we transformed bacteria DH5α by heat-shock, amplified them in the presence of kanamycin, and isolated the plasmid by Qiagen Maxiprep (Qiagen 12163) according to manufacturer’s specifications. A plasmid expressing mouse mitofusin 2 (Mfn2) with an appended C-terminal Myc epitope tag, and parent “empty” plasmid pCLβW [31] were gifts of Dr. David C. Chan (California Institute of Technology). One day before the transfection, 5.0 × 10^5^ cells were seeded on a 4-well Nuc Lab-Tek chambered coverglass. Cells were transfected for 6 h with 1 μg DNA using Lipofectamine 2000 (Invitrogen) according to the manufacturer’s specifications and assayed after 24 h.

### Immunoblotting

To prepare whole-cell extracts, cells were lysed in RIPA cell lysis buffer (Sigma-Aldrich R0278) with protease inhibitor cocktail (Millipore Sigma 11836170001). The lysates were sonicated for 15 s and centrifuged at 12,000 g at 4 °C for 10 min to collect whole-cell protein in the supernatant. Protein concentrations were measured using the Quick Start Bradford Protein Assay Kit (Bio-Rad 5000201) in a Tecan Infinite F200 PRO spectrophotometer. Proteins (10 μg) were resolved on 4–12% Bis-Tris gels (Bio-Rad 161-0375) and transferred to nitrocellulose membranes, followed by Western blotting of the same membranes after stripping with Restore™ PLUS Western Blot Stripping Buffer (Thermo Scientific 46430) for 10 min at RT between each application of antibody. Band densitometry was normalized to vinculin as a loading control. The densitometric values were quantified using ImageJ.

We used the following antibodies: mouse anti-TOM20 (Millipore MABT166), rabbit anti-ACAT1 (Thermo Fisher PA5-34676), rabbit anti-DGAT2 (Abcam ab237613), rabbit anti-HA tag (Cell Signaling 3724), rabbit anti-Myc tag (Abcam ab9106), rabbit anti-PISD (Proteintech 16401-1-AP), rabbit anti-PSS1 (Abcam 157222), and rabbit anti-vinculin (Abcam 129002). For detection we used Alexa Fluor 488 goat anti-rabbit IgG (Invitrogen A-11008) and Alexa Fluor 594 goat anti-mouse IgG (Invitrogen A-11005).

### Live cell imaging

Cells were plated in 4-well Nuc Lab-Tek chambered coverglass imaging plates (Thermo Fisher 155382) followed by transfection with pMAMtracker-Green [30]. Mitochondria and ER in the living cells were visualized after transfection with mitoDsRed (Clontech 632421) and BFP-Sec61β (Addgene 49154). Twenty-four hours post-transfection, images of triple-transfected cells were acquired using a Leica SP5 point scanning confocal microscope (63× objective) equipped with an environmental chamber maintained at 37 °C/5% CO_2_. For image analysis, clusters of cells were identified as regions of interest, and the fluorescence intensity was obtained using Image J software.

### Mitochondrial membrane potential

Mitochondrial polarization state was assessed with tetramethylrhodamine methyl ester (TMRM, Invitrogen T668). TMRM is a cationic fluorophore that sequesters to the matrix of polarized mitochondria but diffuses upon mitochondrial depolarization [32]. Cells were incubated with 20 nM TMRM for 30 min at 37 °C. Cells were then rinsed twice with PBS and resuspended in 200 μl phenol red-free DMEM medium (Thermo Fisher Scientific 12348017). The samples were then processed in a flow cytometer (BD Fortessa), and the median green fluorescence intensity for each sample was determined. Data were analyzed using FCS Express 6 Research Edition (DeNovo Software).

### Seahorse bioenergetic analysis

Mitochondrial respiration in cells was measured using a Seahorse Bioscience XF24 extracellular flux analyzer. Equal numbers of cells (4 × 10^4^/well) were seeded in XF24-well microplates. Before initiation of measurements, cells were rinsed and incubated with XF base medium (Agilent 102353-100) supplemented with 25 mM glucose and 2 mM sodium pyruvate (Thermo Fisher Scientific 11360070), pH 7.4. After 45 min incubation in a CO_2_-free incubator at 37 °C, the oxygen consumption rate (OCR) was measured at the basal state and after sequential injection of 1 μM oligomycin (ATP synthase inhibitor; Sigma-Aldrich 75351), 0.75 μM carbonyl cyanide-p trifluoromethoxyphenylhydrazone (FCCP; uncoupler; Sigma-Aldrich C2920), and 1 mM rotenone/antimycin A (complex I/III inhibitor; Sigma-Aldrich R8875 and A8674, respectively). All OCR values were normalized to cell number after the experiment. The readouts were used to define the bioenergetic parameters as follows: ATP-linked OCR = OCR_Baseline_-OCR_Oligomycin_. Measurements were performed on at least 3 technical replicates and the experiment was repeated at least 4 times (biological replicates). The Wave report generator (Agilent) was used for analysis.

### ETC inhibitors

In order to examine the effect of different inhibitors of the electron transport chain (ETC) on MAM function, we exposed ρ^+^ cells to pharmacological inhibitors of the individual ETC components, followed by measurement of phospholipid transport/synthesis. The inhibitors used were rotenone (1 μM), an inhibitor of complex I; atpenin A5 (20 nM), an inhibitor of complex II [33], antimycin-A (1 μM), an inhibitor of complex III; cyanide (50 μM), an inhibitor of complex IV and oligomycin (1 μM), an inhibitor of complex V. In addition, we used two uncouplers (FCCP and BAM15 [34]; both 0.75 μM). Cells were exposed to the inhibitors for 6 h, after which the synthesis of PtdSer and PtdEtn was measured as described above.

### Measurement of cell viability, cytotoxicity, and apoptosis

To determine cell viability, cytotoxicity, and apoptosis, the ApoTox-Glo Triplex Assay (Promega, Madison, WI, USA) was used. The test measures live-cell protease activity using a fluorogenic, cell-permeant peptide substrate (GF-AFC substrate), dead-cell protease activity using a cell-impermeant, fluorogenic peptide substrate (bis-AAF-R110 substrate), and caspase-3/7 activation as a key indicator of apoptosis. Cells were seeded in black 96-well half-area plates with clear bottom. The assay was performed according to the manufacturer’s instructions. Measurements were performed on at least 5 technical replicates and the experiment was repeated at least 3 times (biological replicates).

### Statistical analysis

Results were expressed as mean ± SD (standard deviation) unless stated otherwise. Two-tailed Student’s test was used to compare two different conditions using GraphPad Prism software v 7.0. An asterisk indicates significant differences (*p < 0.05; **p < 0.01; ***p < 0.0001; ns, not significant).

## Results

### MAM function is disrupted in ρ^0^ cells

**Fig. 1:**
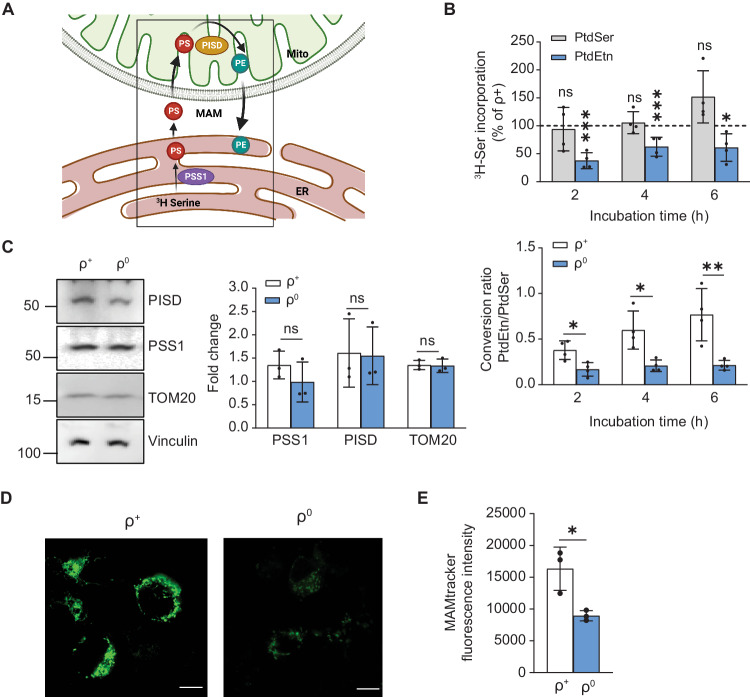
MAM function in ρ^0^ cells: role of phospholipid transfer. **A** Schematic representation of phospholipid synthesis/transport at the MAM. Note that both ER and mitochondria are involved in this process. PISD, phosphatidylserine decarboxylase; PSS1, phosphatidylserine synthase 1; PS, phosphatidylserine; PE, phosphatidylethanolamine; Mito, mitochondria. **B** Conversion of ^3^H-Ser into ^3^H-PtdSer and ^3^H-PtdEtn in ρ^0^ cells relative to that in control ρ^+^ cells (dotted line) for the indicated times (*n* = 4 independent experiments). Note the severe drop in PtdEtn synthesis in ρ^0^ vs ρ^+^ cells, whereas that of PtdSer was unchanged. Quantification of the ratio of PtdEtn/PtdSer in ρ^+^ and ρ^0^ cells analyzed in B below. Note the decrease in the conversion of ^3^H-PtdSer to ^3^H-PtdEtn in ρ^0^ cells. **C** Representative Western blot of phospholipid synthesis-related proteins (PSS1 and PISD), and of TOM20 (a mitochondrial marker), relative to vinculin in ρ^+^ and ρ^0^ cells. 20 μg protein loaded/lane. Molecular weight markers at left, in kDa. Quantitation at right. Note similar protein levels in the two cells. **D** Representative confocal microscopy images of MAM (MAMtracker-Green, green). Scale bars = 10 μm. Note the increase in MAMtracker fluorescence intensity in ρ^0^ cells compared to ρ^+^ cells. **E** Quantitation of the fluorescence intensity of MAMtracker-Green in transfected ρ^+^ and ρ^0^ cells (*n* = 4 independent experiments, examining 7-8 transfected cells in each experiment). Data here and in all other figures are expressed as mean ± SD. Statistical significance was analyzed by Student’s t-test (*, *p* < 0.05; **, *p* < 0.01; ***, *p* < 0.0001; ns, not significant).

We first assessed MAM function in the most extreme case of bioenergetic failure, namely human ρ^0^ cells. These cells are completely devoid of mtDNA [35] and therefore have no functioning respiratory chain (see Supplementary Fig. 1**)**. To do this we first assessed the synthesis and transport of phospholipids (PLs), a well-recognized measure of MAM function [36]. Briefly, PtdSer is synthesized in the MAM by phosphatidylserine synthases (PSS1 [gene *PTDSS1*] and PSS2 [gene *PTDSS2*]) [37]; PtdSer then translocates to the mitochondria where it is decarboxylated by phosphatidylserine decarboxylase (PISD) [38] to form PtdEtn; PtdEtn then travels back to the MAM, where it traffics to the rest of the cell or undergoes further modifications [38] (see Fig. 1A). Accordingly, we measured the incorporation of radiolabeled L-serine (^3^H-Ser) into newly-synthesized PtdSer and PtdEtn at different time intervals (2, 4, and 6 h) in ρ^0^ and control respiratory-competent ρ^+^ cells. We found that ^3^H-PtdSer synthesis in ρ^0^ cells was essentially identical to that in ρ^+^ cells (Fig. 1B), whereas ^3^H-PtdEtn synthesis was reduced significantly (Fig. 1B and Supplementary Fig. 2A), as was the ratio of PtdEtn/PtdSer, a measure of conversion efficiency: ρ^+^ cells converted ~30% of their nascent ^3^H-PtdSer to ^3^H-PtdEtn at 2 h, ~50% at 4 h, and ~70% at 6 h, whereas ρ^0^ cells converted less than 30% independently of the time point (Fig. 1B). The reduction in PL synthesis was in fact due to the MAM defects, since the enzymes involved in this process (PSS1, PISD), and mitochondrial mass (TOM20), as measured by Western blot, remained unaltered in both cells (Fig. 1C).

The deficient synthesis of PtdEtn in ρ^0^ cells prompted us to investigate whether ER-mitochondrial contacts were physically altered. We therefore transfected the cells with MAMtracker-Green to detect MAM (in green) by confocal microscopy [30]. Note that MAMtracker-Green emits fluorescence only when the ER and the mitochondria are in close proximity, and does so reversibly [30]; the reporter was expressed equally in both cells (Supplementary Fig. 2B). We found that ρ^0^ cells exhibited a significant reduction in MAMtracker fluorescence intensity (~50% of that in ρ^+^ cells (Fig. 1D, E). Our data suggest that in ρ^0^ cells, PtdSer synthesis is unimpaired but that the transfer of PtdSer to mitochondria for conversion to PtdEtn is reduced, as a consequence of a lower degree of physical association between ER and mitochondria.

**Fig. 2:**
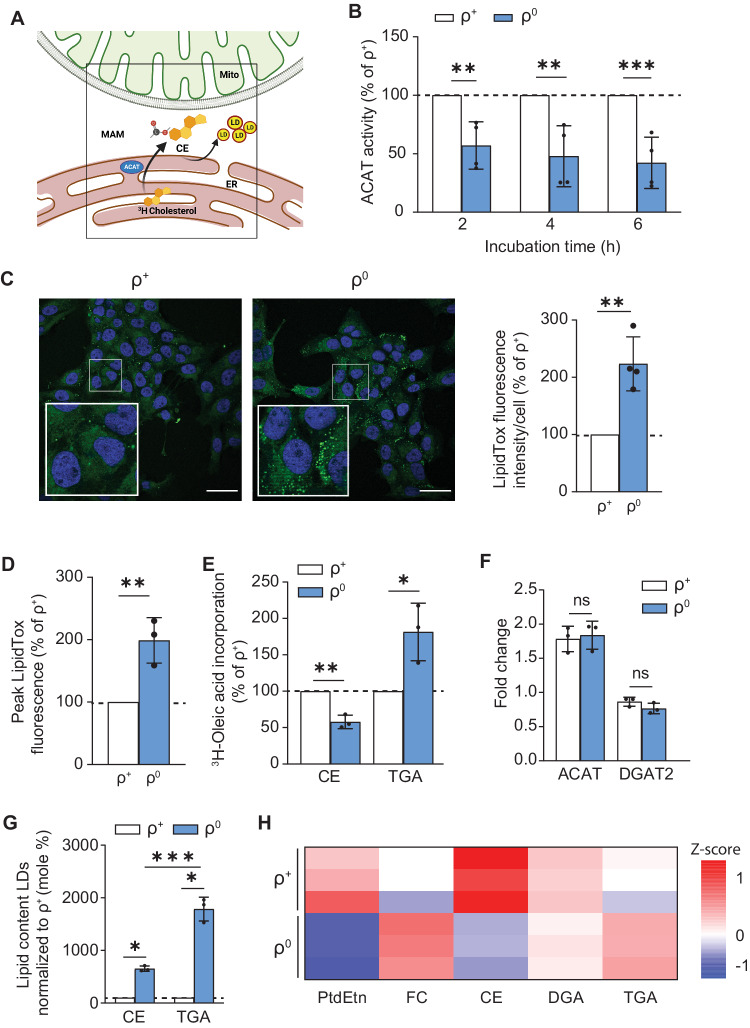
MAM function in ρ^0^ cells: role of cholesteryl ester synthesis. **A** Schematic representation of CE synthesis at the MAM. Note that MAM, but not mitochondria, is involved in this reaction. ACAT, acyl-CoA:cholesterol acyltransferase 1; CE, cholesteryl ester; LD, lipid droplet. **B** Conversion of ^3^H-cholesterol to ^3^H-CE (a MAM-specific function) in ρ^0^ cells relative to ρ^+^ cells (dotted line) for the indicated times (*n* = 3 independent experiments). Note that the conversion of cholesterol in CE was reduced in ρ^0^ compared to ρ^+^ cells. **C** Representative confocal microscopy images of lipid droplet staining with LipidTox Green (green), and nuclei labeled with DAPI (blue), in ρ^+^ and ρ^0^ cells. Scale bars = 45 μm. Expanded images in boxes. Quantitation of fluorescence intensity of LipidTox Green in ρ^0^ cells relative to ρ^+^ cells (dotted line) at right (*n* = 4 independent experiments with >50 cells in each experiment). Note increase in LD formation in ρ^0^ cells. **D** Quantitation of fluorescence intensity of LipidTox Green in ρ^0^ cells relative to ρ^+^ cells (dotted line) by flow cytometry (*n* = 3 independent experiments with >20000 cells in each experiment). **E** Conversion of ^3^H-oleic acid to ^3^H-cholesteryl oleate (CE) and ^3^H-triglycerides (TGA) in ρ^0^ cells relative to ρ^+^ cells (dotted line) after 4 hours. Note the increase in the levels of TGA in ρ^0^ cells and the decrease in CE, consistent with the decrease in ^3^H-CE in panel B (*n* = 3 independent experiments). **F** Quantitation of LD synthesis-related proteins ACAT1 and DGAT2 relative to vinculin in ρ^+^ and ρ^0^ cells (*n* = 3). Note similar protein levels in the two cells. **G** Quantitation of the lipid content in isolated LDs from ρ^0^ cells relative to ρ^+^ cells (dotted line) as measured by lipidomics (*n* = 3 independent experiments). Note the increase in TGA compared to CE in ρ^0^ cells, consistent with the increase in ^3^H-TGA in (**E**). **H** Heatmap representation of the lipidomics analysis of crude mitochondria (containing MAM), focusing on PtdEtn, free cholesterol (FC), cholesteryl esters (CE), diacylglycerides (DGA) and triglycerides (TGA) in ρ^+^ and ρ^0^ cells (*n* = 3). Results are expressed as Z-scores.

We measured a second, independent, MAM function, namely cholesteryl ester synthesis. MAM is enriched in a key cholesterol-metabolism enzyme, acyl-CoA:cholesterol acyltransferase 1 (ACAT1; gene *SOAT1*), that catalyzes the conversion of free cholesterol to cholesteryl esters (CEs) [27, 39] (Fig. 2A). We therefore incubated ρ^+^ and ρ^0^ cells with ^3^H-cholesterol and tracked its conversion to ^3^H-CE by quantitative thin layer chromatography (TLC) [13, 17]. We found that ACAT1 activity (i.e formation of CEs) was downregulated significantly (by ~50%) in the ρ^0^ cells compared to ρ^+^ cells (Fig. 2B). As expected, the level of free cholesterol (which comprises the great majority of the total pool of cholesterol species [40] was unchanged (Supplementary Fig. 2C).

Elevated ACAT1 activity (i.e., CE production) typically results in the deposition of those CEs in lipid droplets (LDs) [41]. We therefore stained the cells with LipidTox Deep Green neutral lipid stain and quantified the LDs using confocal microscopy and flow cytometry. We found that the number (Fig. 2C) and fluorescence intensity (Fig. 2D and Supplementary Fig. 2D) of lipid droplets was higher (up to 2-fold) in ρ^0^ cells compared to ρ^+^ cells, similar to the findings of others [42].

In addition to CEs, lipid droplets store triglycerides (TGAs) upon an increase in intracellular free fatty acid (FFA) levels [41]. We therefore asked whether the increased lipid droplet intensity was due to an increased in TGA content in LDs. Accordingly, we measured the activity of diacyglycerol O-acyltransferase 2 (DGAT2), a MAM-localized enzyme [43] that converts diacylglycerols to TGAs, by incubating the cells with ^3^H-oleic acid and measuring its conversion into ^3^H-TGAs. We found an increase in TGA synthesis in ρ^0^ cells compared to ρ^+^ cells (Fig. 2E). On the other hand, we found reduced production of CEs (as ^3^H-cholesteryl oleate) (Fig. 2E and Supplementary Fig. 2E), consistent with the decrease in ACAT activity shown above (Fig. 2B). As before, despite of the decrease in CEs and the increase in TGAs in ρ^0^ cells, the levels of these two enzymes (ACAT1 and DGAT2) were unchanged compared to the level in ρ^+^ cells (Fig. 2F and Supplementary Fig. 2F). In addition, lipidomic analysis of isolated LDs revealed that the total TGA content in ρ^0^ cells was 3-fold higher than that of total CEs (Fig. 2G).

Additionally, we analyzed the lipid composition of the crude mitochondrial fraction (containing MAM), in order to identify a potential lipid signature specific to ρ^0^ cells. Consistent with our observations in radiolabeled whole cells, the pool of PtdEtn in the ρ^0^ cells decreased (Fig. 2H). In addition, we found a decrease in the CE levels, and an increase in DGA/TGA species (containing 16- and 18-carbon-long saturated and monounsaturated fatty acyl chains), generally associated with the activation of lipogenic pathways [44] (Fig. 2H).

Taken together, these results suggest that MAM disruption induces an inverse relationship in the synthesis of CEs and TAGs, two lipid species involved in the mobilization of lipids in the cell, and support the notion that MAM is intimately involved in this process.

### MAM function is disrupted in cells harboring mutations causing human mitochondrial disease

We hypothesized that MAM may also be affected in cells containing pathogenic mutations causing mitochondrial disease. We first focused on mutations in mtDNA. Specifically, we analyzed MAM function in isogenic pairs of cybrids (i.e., homoplasmic wild-type (WT) and mutant) derived from patients with two quite different mitochondrial diseases, Kearns-Sayre syndrome (KSS) and maternally-inherited Leigh syndrome (MILS) [22].

**Fig. 3:**
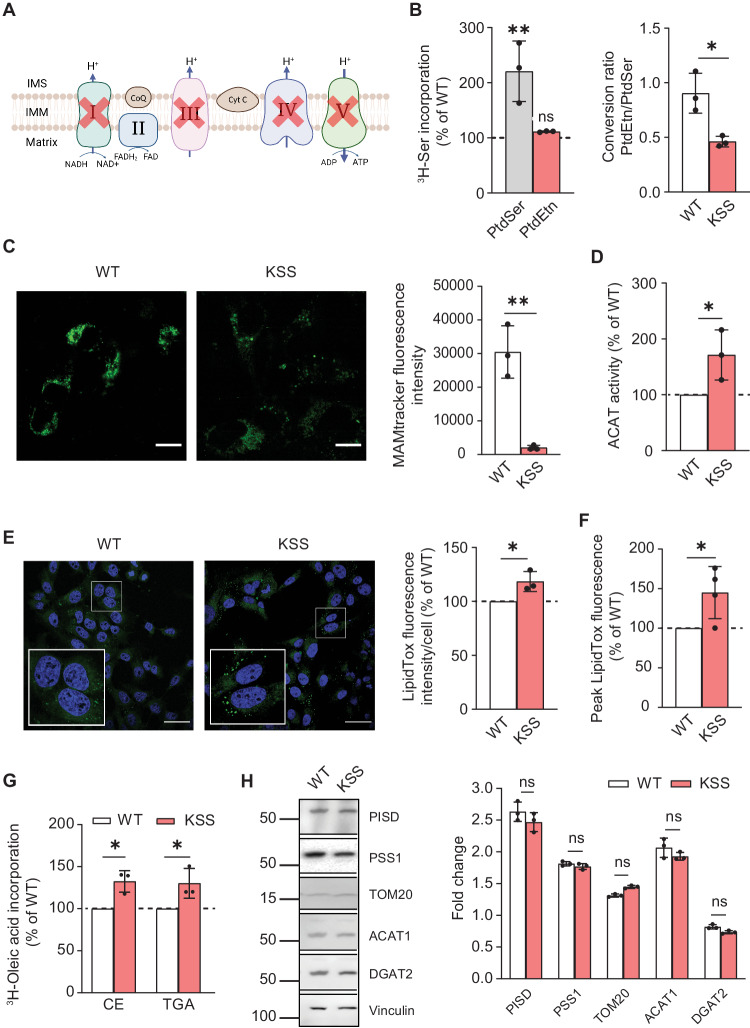
Analysis of MAM function in KSS cybrids. **A** Schematic representation of the R.C. complexes compromised in KSS (red X’s). **B** Incorporation of ^3^H-Ser into ^3^H-PtdSer and ^3^H-PtdEtn in Δ-KSS cybrids compared to WT-KSS cybrids (dotted line) at 4 h (*n* = 3). Quantitation of the ratio of PtdEtn/PtdSer in WT-KSS and Δ-KSS cybrids analyzed in B at right. Note the decrease in the conversion of ^3^H-PtdSer to ^3^H-PtdEtn in Δ-KSS cybrids, similar to what was observed in ρ^0^ cells. **C** Representative confocal microscopy images of MAM (MAMtracker-Green, green) in WT-KSS and Δ-KSS cybrids. Scale bars = 15 μm. Quantification of MAMtracker-Green fluorescence intensity in transfected WT-KSS and Δ-KSS cybrids, as in Fig. 1E. **D** Conversion of ^3^H-cholesterol to ^3^H-CE in Δ-KSS relative to WT-KSS cybrids (dotted line) at 4 h (*n* = 3). Note that the decrease in ACAT activity in Δ-KSS was opposite to what we observed in ρ^0^ cells. **E** Representative confocal microscopy images of lipid droplet formation staining with LipidTox Green (green), and nuclei labeled with DAPI (blue), in WT-KSS and Δ-KSS cybrids. Scale bars = 45 μm. Expanded images in boxes. Quantitation of LipidTox Green fluorescence intensity as in Fig. 2C. **F** Quantitation of LipidTox Green fluorescence intensity in Δ-KSS cybrids compared to WT-KSS cybrids (dotted line) by flow cytometry as in Fig. 2D. Note increase in LDs, consistent with the CE data shown in panel **D**. **G** Conversion of ^3^H-oleic acid to ^3^H-cholesteryl oleate (CE) and ^3^H-triglycerides (TGA) in Δ-KSS cybrids compared to that in WT-KSS cybrids (dotted line) at 4 h (*n* = 3). Note that Δ-KSS cells accumulate both lipid species. **H** Representative Western blot of phospholipid synthesis-related proteins (PSS1 and PISD), LD-related proteins (ACAT1 and DGAT2), and mitochondria (TOM20), as in Fig. 1C. No change at the proteins level were observed.

The KSS mutation was a ∼1.9-kb partial deletion of mtDNA (Δ-mtDNA) and homoplasmic cells containing this mutation have no functioning respiratory chain (see Supplementary Fig. 1) [23, 45]. Deletions in KSS typically remove some R.C. complex subunit genes as well as critical tRNA genes that are absolutely required for translation of all 13 mtDNA-encoded OxPhos mRNAs and hence, for ATP synthesis (see Fig. 3A) [7]. Analysis of phospholipid synthesis in homoplasmic mutant (Δ-KSS) cybrids showed that PtdSer synthesis at 4 h increased significantly compared to that in isogenic homoplasmic wild-type (WT-KSS) cybrids derived from the same patient, whereas PtdEtn levels were essentially unchanged (Fig. 3B and Supplementary Fig. 3A). Notably, the turnover of PtdEtn/PtdSer was significantly lower in the Δ-KSS cybrids, with only ~50% conversion of PtdSer to PtdEtn in Δ-KSS cells compared to almost 90% in WT-KSS cells (Fig. 3B), suggesting a phospholipid transport defect. Moreover, Δ-KSS cybrids exhibited a significant decrease in MAMtracker-Green intensity (Fig. 3C).

With respect to ACAT activity, we found a significant increase in CE synthesis in Δ-KSS cybrids at 4 h (∼2-fold over WT-KSS) (Fig. 3D and Supplementary Fig. 3B), and observed numerous LDs in Δ-KSS, but not in WT-KSS, cells (Fig. 3E, F, and Supplementary Fig. 3C). The incorporation of ^3^H-oleic acid into both CE and TGAs was increased significantly in Δ-KSS cells (Fig. 3G), indicating that in this disorder both lipid species are perturbed. As in the ρ^0^ cells, these changes were not due to reduced enzyme levels (Fig. 3H).

Taken together, these findings suggest that ER-mitochondrial connectivity is impaired in Δ-KSS cybrids, similar to what we found in ρ^0^ cells.

**Fig. 4:**
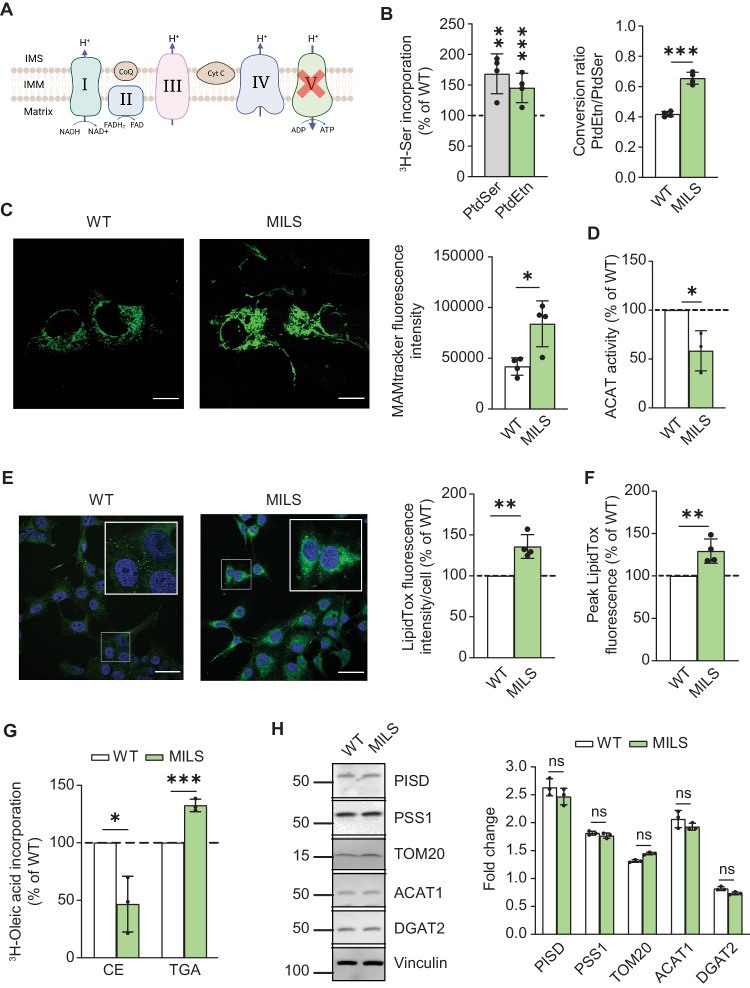
Analysis of MAM function in MILS cybrids. **A** Schematic representation of the T8993G mutation in ATPase6 that causes NARP and MILS. In contrast to KSS, in these cells the respiratory chain is intact and is essentially unaffected; only ATP synthesis is compromised. **B** Left: Incorporation of ^3^H-Ser into ^3^H-PtdSer and ^3^H-PtdEtn in MILS cybrids compared to WT-MILS cybrids (dotted line) at 4 h (*n* = 4). Note that in contrast to what we saw in KSS (Fig. 3B), both PtdSer and PtdEtn synthesis were significantly increased in MILS cybrids. Right: Quantitation of the ratio of PtdEtn/PtdSer in WT-MILS and MILS cybrids. Note increase in the conversion of ^3^H-PtdSer to ^3^H-PtdEtn in MILS cybrids. **C** Representative confocal microscopy images of MAM (MAMtracker-Green, green) in WT-MILS and MILS cybrids. Scale bars = 15 μm. Quantification of MAMtracker-Green fluorescent intensity in transfected WT-MILS and MILS cybrids (*n* = 4 independent experiments with 7-8 transfected cells in each experiment) at right. Note increase in MAMtracker fluorescence intensity, in agreement with the biochemical data shown in (**B**). **D** Conversion of ^3^H-cholesterol to ^3^H-CE in MILS relative to WT-MILS cybrids (dotted line) at 4 h (*n* = 3). Note that MILS cells mimic the CE results seen in ρ^0^ cells (Fig. 2B). **E** Representative confocal microscopy images of lipid droplet staining with LipidTox Green (green), and nuclei labeled with DAPI (blue), in WT-MILS and MILS cybrids. Scale bars = 45 μm. Expanded images in boxes. Quantitation at right, as in Fig. 2C. **F** Quantitation of fluorescence intensity of LipidTox Green in MILS cybrids compared to WT-MILS cybrids (dotted line) by flow cytometry, as in Fig. 2D. Note increase in LDs, in contrast with the CE data shown in (**D**). **G** Conversion of ^3^H-oleic acid to ^3^H-cholesteryl oleate (CE) and ^3^H-triglycerides (TGA) in MILS cybrids compared to WT-MILS cybrids (dotted line) at 4 h (*n* = 3). Note that MILS cells accumulate TGA but not CE. **H** Representative Western blot of phospholipid- and LD-related proteins, as in Fig. 3H. No changes at the protein level were observed.

We next focused on cybrids from a patient with the T8993G mutation in ATPase6 that causes neuropathy, ataxia, and retinitis pigmentosa (NARP) at relatively low mutant loads, and MILS at high mutant loads [7]. Importantly, and in contrast to KSS, in these cells the respiratory chain is intact and unaffected; only ATP synthesis is compromised (see Fig. 4A and Supplementary Fig. 1B) [46]. Notably, in contrast to what we saw in KSS, both PtdSer and PtdEtn synthesis were significantly *increased* (∼1.4-fold at 6 h) in homoplasmic mutant MILS cybrids compared to WT-MILS cybrids, and converted more PtdSer to PtdEtn (Fig. 4B and Supplementary Fig. 4A). Furthermore, the degree of ER-mitochondrial apposition, as measured by MAMtracker-Green, was significantly higher (by ~50%) in MILS compared to WT-MILS cybrids (Fig. 4C). When we measured ACAT activity, we found that CE synthesis in MILS cybrids decreased significantly (by ~40% at 6 h) (Fig. 4D and Supplementary Fig. 4B), but we observed a significant increase in LD formation (Fig. 4E, 4F and Supplementary Fig. 4C) that was likely the result of an accumulation of TGAs, not CEs (Fig. 4G). As before, there were no changes in relevant enzyme protein levels (Fig. 4H).

Taken together, in contrast to what we observed in KSS and ρ^0^ cells, we found an increase in the physical association between ER and mitochondrial in MILS cells, even though ATP synthesis is impaired in both disorders.

**Fig. 5:**
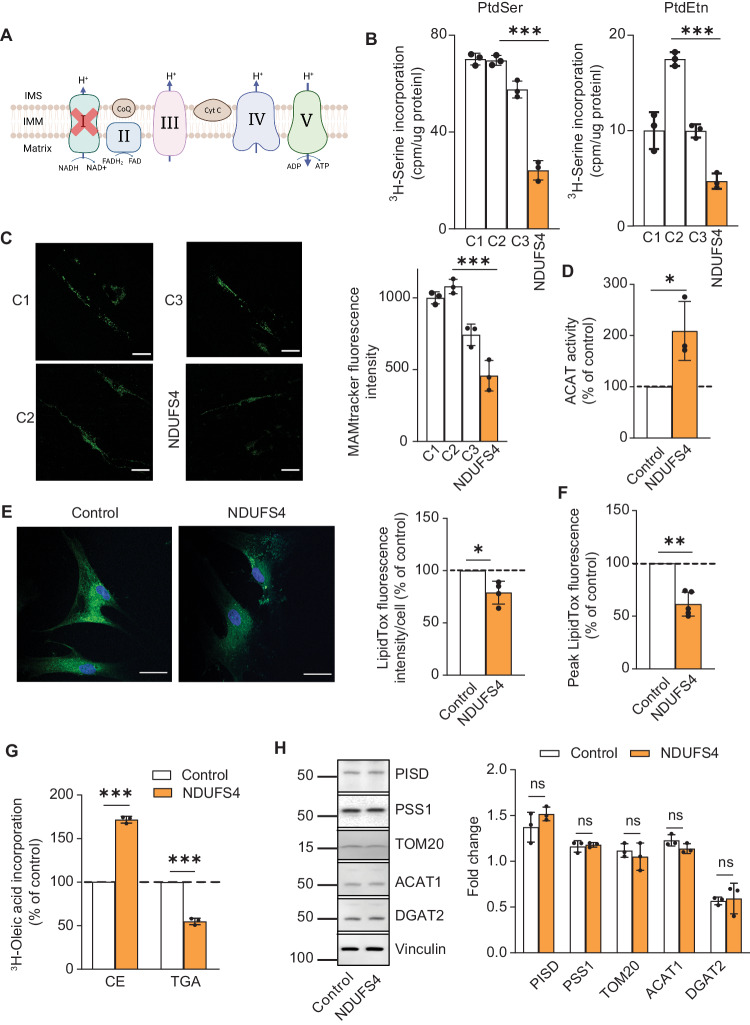
Analysis of MAM function in NDUFS4-mutant fibroblasts. **A** Schematic representation of the mutation in the nucleus-encoded NDUFS4 subunit of complex I. **B** Incorporation of ^3^H-Ser into ^3^H-PtdSer and ^3^H-PtdEtn in control fibroblasts (C1, WT001; C2, KR003; C3, FC8) and NDUFS4 fibroblasts at 4 h (*n* = 3). Left: Note that both PtdSer and PtdEtn synthesis were significantly decreased in mutant fibroblasts. Right: Quantification of the ratio of PtdEtn/PtdSer. Note that no change in the conversion efficiency of ^3^H-PtdSer to ^3^H-PtdEtn was observed in mutant fibroblasts. **C** Representative confocal microscopy images of MAM (MAMtracker-Green, green) in controls and NDUFS4 fibroblasts. Scale bars = 15 μm. Quantitation at right, as in Fig. 2C. Note decrease in MAMtracker fluorescence intensity, in agreement with the biochemical data shown in panel B. **D** Conversion of ^3^H-cholesterol to ^3^H-CE in NDUFS4 fibroblasts relative to control (dotted line) at 4 h (*n* = 3). **E** Representative confocal microscopy images of lipid droplet staining with LipidTox Green (green), and nuclei labeled with DAPI (blue), in control and NDUFS4 fibroblasts. Scale bars = 45 μm. Quantification at right, as in Fig. 2C. Note decrease in LD formation. **F** Quantitation of fluorescence intensity of LipidTox Green in NDUFS4 fibroblasts relative to control (dotted line), as in Fig. 2D. **G** Conversion of ^3^H-oleic acid into ^3^H-cholesteryl oleate (CE) and ^3^H-triglycerides (TGA) in mutant NDUFS4 fibroblasts compared to control (dotted line) at 4 h (*n* = 3). Note that mutant fibroblasts accumulate only CE, consistent with the biochemical CE data shown in (**D**). **H** Representative Western blot as in Fig. 3H. No changes in the protein levels were observed.

Finally, we asked whether mutations in nucleus-encoded R.C. genes have similar effects on MAM function. We therefore measured MAM function in human fibroblasts containing a mutation (355 G > A; p.Asp119His) in the nucleus-encoded NDUFS4 subunit of complex I [25] (see Fig. 5A), and in which ATP production is compromised (Supplementary Fig. 1B). The analysis cohort consisted of a patient (NDUFS4, female, age 3) and 3 independent healthy individuals as controls (C1, female, age 3; C2, female, age 3; C3, female, age 35) (Supplementary Fig. 1A). In the phospholipid assay, we found a moderate decrease in PtdSer and PtdEtn synthesis in NDUFS4 fibroblasts compared to control fibroblasts (up to 60% reduction in both species at 6 h) (Fig. 5B and Supplementary Fig. 5A) and a correspondingly moderate ( ~ 50%) decrease in ER-mitochondrial apposition as measured by MAMtracker-Green (Fig. 5C). Notably, the 3 control fibroblasts used in this study exhibited a similar trend compared to NDUFS4 fibroblasts, consistent with the findings of the mitochondrial respiratory assay (Supplementary Fig. 1B), implying that the results were independent of the age of the donor. Thus, for the following experiments, we used one control (FC8) as a representative sample. In the ACAT assay we observed significantly higher CE synthesis in the patient cells (up to ∼2-fold over control) (Fig. 5D and Supplementary Fig. 5B) but decreased LD formation (∼2-fold lower signal than in control) (Fig. 5E, F, and Supplementary Fig. 5C). Moreover, the incorporation of ^3^H-oleic acid into ^3^H-TGA decreased, whereas the incorporation of ^3^H-cholesterol into ^3^H-CE increased (Fig. 5G). The cause of this unusual lipid pattern in complex I deficiency is currently unknown. As before, we detected no changes in relevant enzyme protein levels (Fig. 5H). Taken together, these data suggest that, although the specific details vary, mutations in nucleus-encoded R.C. subunits alter MAM-mediated lipid metabolism, and that OxPhos-induced MAM dysfunction is independent of the origin of the mutation (i.e., mtDNA- vs. nDNA).

### Analysis of MAM function following pharmacological inhibition of OxPhos complexes

**Fig. 6:**
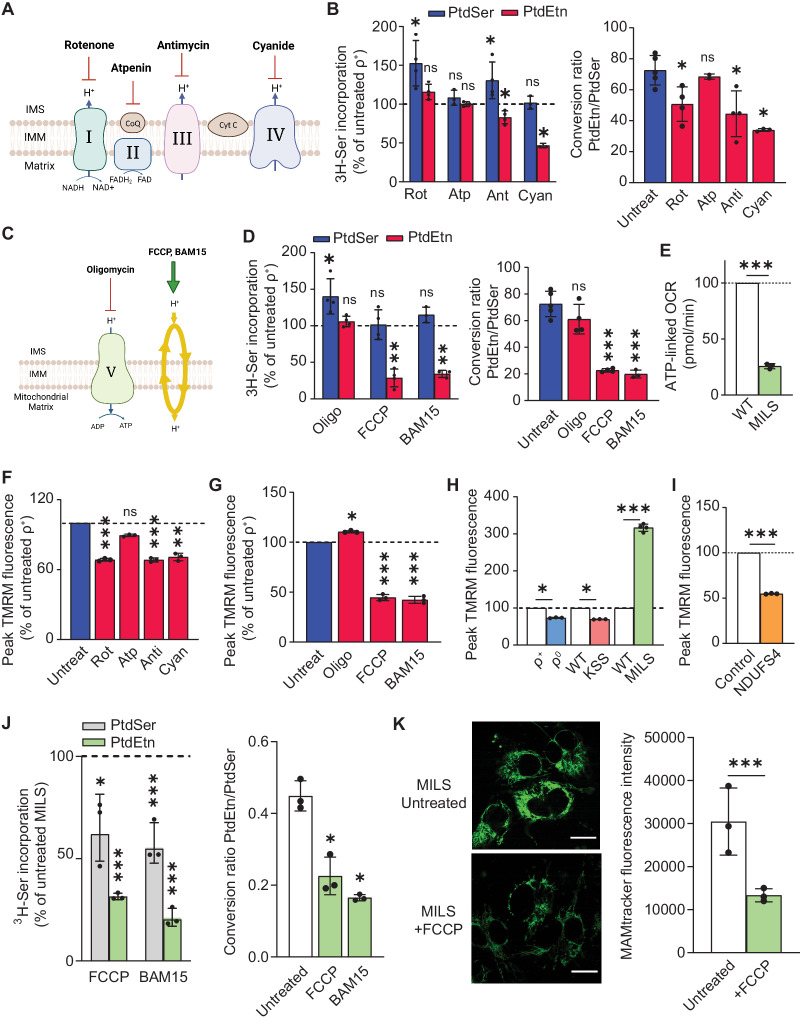
MAM function in R.C. complexes: the role of membrane potential. **A** Schematic representation of the specific R.C. inhibitors used in the present study. **B** Incorporation of ^3^H-Ser into ^3^H-PtdSer and ^3^H-PtdEtn in 143B (ρ^+^) cells exposed to the indicated inhibitors compared to untreated cells (dotted line) at 6 h (*n* = 3). Quantitation of PtdEtn/PtdSer at right. Note reduction in phospholipid transport after inhibition of CI, CIII, and CIV, but not of CII. **C** Schematic representation of the specific OxPhos inhibitors (oligomycin for CV) and uncouplers (FCCP and BAM15) used here. **D** Incorporation of ^3^H-Ser into ^3^H-PtdSer and ^3^H-PtdEtn in ρ^+^ cells exposed to oligomycin and uncouplers (dotted line) at 6 h (*n* = 3). Quantification of PtdEtn/PtdSer at right. **E** Quantification of ATP-linked OCR in WT-MILS and mut-MILS cybrids (*n* = 3). Note the decrease in ATP production in mut-MILS cybrids. **F** Quantitation of the mitochondrial membrane potential (MMP) measured by TMRM after exposing ρ^+^ cells to the R.C. inhibitors (*n* = 3). Note that inhibition of complexes I, III, and IV induced a reduction of MPP compared to untreated cells, whereas inhibition of complex II had little effect. **G** Quantitation of MMP after exposing ρ^+^ cells to oligomycin and to uncouplers (*n* = 3). Note that inhibition of complex V induced an increase in MMP compared to untreated cells, whereas both uncouplers induced mitochondrial depolarization. **H** Quantitation of MMP in the cells and cybrids studied here (*n* = 3). Note that ρ^0^ cells and KSS cybrids (both with essentially no respiratory chain function) maintained lower MMP, whereas MILS cybrids (with complex V affected, but with an intact respiratory chain) exhibited a higher MMP, consistent with the pharmacological inhibition of OxPhos complexes, as shown in panels F and G. **I** Quantitation of MMP in control and NDUFS4 fibroblasts (*n* = 3). Note the decrease in MMP. **J** Incorporation of ^3^H-Ser into ^3^H-PtdSer and ^3^H-PtdEtn in mut-MILS cybrids exposed to uncouplers compared to that in untreated mut-MILS cybrids at 6 h (*n* = 3). Quantitation of PtdEtn/PtdSer at right. Note the significant reduction in MPP in MILS cells exposed to the uncouplers. **K** Representative confocal microscopy images of MAM (MAMtracker-Green, green) in mut-MILS cells untreated or treated with FCCP. Scale bars = 15 μm. Quantitation at right, as in Fig. 2E. Note decrease in MAMtracker fluorescence intensity, in agreement with the uncoupling data shown in (**J**).

In order to further understand the OxPhos-MAM relationship in the mitochondrial diseases described above, and to provide insight into potential mechanisms, we asked whether inhibition of specific respiratory chain complexes affect ER-mitochondria communication. We therefore exposed ρ^+^ cells to specific R.C. inhibitors (rotenone for CI; atpenin A5 for CII; antimycin for CIII, and cyanide for CIV) for 6 h (i.e., 2 h pre-incubation in Ser-free medium containing the inhibitor followed by 4 h in medium containing ^3^H-Ser and the inhibitor) and measured MAM parameters (see Fig. 6A). We observed phospholipid transport defects in cells treated with rotenone, antimycin, and cyanide compared to untreated cells (Fig. 6B): the conversion of PtdSer to PtdEtn was reduced moderately but significantly (by ~25-40%), suggesting an accumulation of PtdSer and/or a lack of its conversion to PtdEtn, presumably as a result of a decrease in ER-mitochondria connectivity. Notably, inhibition of complex II with atpenin A5 did not affect phospholipid synthesis (Fig. 6B). Overall, these findings imply, but of course do not prove, that deficiency of the R.C. complexes that are involved in proton pumping (i.e., I, III, and IV) compromise ER-mitochondrial connectivity, whereas deficiency of complex II, which does not pump protons, does not seem to have this effect, suggesting that the R.C. proton-pumping activity participates in the regulation of the ER-mitochondrial connectivity.

To explore this issue further, and to identify factors that perturb the OxPhos-MAM axis, we asked whether mitochondria-derived ATP is required for ER-mitochondria communication. To do this, we inhibited OxPhos by treating ρ^+^ cells with oligomycin (to inhibit ATP synthase, or CV) and with the uncouplers FCCP and BAM15 (which “short-circuits” electron transport) (schematic shown in Fig. 6C) and measured phospholipid synthesis. Interestingly, in cells treated with oligomycin, the levels of PtdSer increased moderately but significantly (by ~40%), whereas PtdEtn levels were unaffected relative to untreated cells (Fig. 6D), and there was no significant change in the ratio of PtdEtn/PtdSer (Fig. 6D). In contrast, ρ^+^ cells treated with the uncouplers showed no change in ^3^H-PtdSer synthesis but a profoundly decreased synthesis of ^3^H-PtdEtn relative to untreated cells (∼65% for both FCCP and BAM15 [Fig. 6D]). These results imply that reductions in mitochondria-derived ATP alone cannot explain the altered ER-mitochondrial connectivity in OxPhos-compromised cells, as ER-mitochondrial communication was up in MILS cells even though ATP levels were down by ~75% (Fig. 6 and Supplementary Fig. 1B).

### Alterations in ER-mitochondrial communication are dependent on membrane potential

While ATP levels appear to play a minor role in MAM dysfunction, we hypothesized that other factors may compromise MAM behavior. Considering that the inhibition of complexes I, III, and IV resulted in a moderate decrease in the ER-mitochondrial connectivity ( ~ 25–40%, Fig. 6B), and that this effect was more pronounced by the uncouplers FCCP and BAM15 (∼65%; Fig. 6C) in ρ^+^ cells, we asked whether the mitochondrial membrane potential (MMP; Δψ) might play a role in regulating ER-mitochondrial communication. To test this, we measured the MMP using tetramethyl rhodamine ester (TMRM) after exposing ρ^+^ cells to the inhibitors described above. Notably, inhibition of complexes I, III, and IV induced a significant reduction of the median peak TMRM fluorescence intensity (31%, 36% and 35%, respectively) compared to untreated cells, whereas inhibition of complex II had little effect (only a slight decrease of 9%) (Fig. 6F). Moreover, inhibition of complex V with oligomycin led to mitochondrial hyperpolarization, as evidenced by a 28% increase in TMRM fluorescence intensity compared to untreated cells (Fig. 6G). In addition, compared to control cells, uncoupling ρ^+^ mitochondria with both FCCP and BAM15 induced mitochondrial depolarization, with a reduction of ~65% in peak TMRM fluorescence intensity (Fig. 6G). Taken together, the effects of the chemical inhibitors on membrane potential (Fig. 6F, G) mirror to a remarkable degree the effects of these inhibitors on MAM function as measured by the phospholipid assay (Fig. 6B, D). In particular, both sets of results suggest that lower MMP disrupts ER-mitochondrial connectivity, whereas higher MMP enhances it, supporting the view that mitochondrial membrane potential plays an important role in regulating ER-mitochondrial communication.

A similar conclusion may be drawn from our analyses of the patient cybrids. Specifically, ρ^0^ cells and KSS cybrids (with no respiratory chain function (Supplementary Fig. 1B)) [22], maintained lower peak TMRM values (Fig. 6H) and reduced MAM connectivity (Figs. 1 and 3, respectively) compared to their WT counterparts. On the other hand, MILS cybrids (with complex V affected, but with an intact respiratory chain) exhibited a higher peak TMRM value (Fig. 6H) and enhanced MAM connectivity (Fig. 4). In addition, NDUFS4 fibroblasts (with complex I affected) showed a lower TMRM fluorescence compared to control, similar what we found in ρ^0^ cells and KSS cybrids (Fig. 6I).

If higher MMP leads to increased ER-mitochondrial communication, then decreased MMP should have the opposite effect. To test this, we exposed MILS cybrids (with reduced MMP and PtdSer/PtdEtn synthesis elevated over WT) to uncouplers, and evaluated MAM function using the PL assay. In agreement with our hypothesis, the uncouplers triggered a decrease of ∼50% in the synthesis of PtdSer and of ∼75% of PtdEtn compared to untreated MILS cybrids (Fig. 6J). Concomitantly, the ratio of PtdEtn/PtdSer in treated cells was reduced by ~50–60% relative to untreated cells (Fig. 6J), indicating a lower degree of ER-mitochondrial communication following the reduction in MMP. Notably, we observed a corresponding decrease ( ~ 60%) in ER-mitochondrial apposition in MILS cybrids treated with FCCP (Fig. 6K).

Overall, these findings suggest that the MMP correlates with the degree of ER-mitochondrial connectivity in these cells.

### Increased ER-mitochondrial communication reverses the deficiencies in MMP

**Fig. 7:**
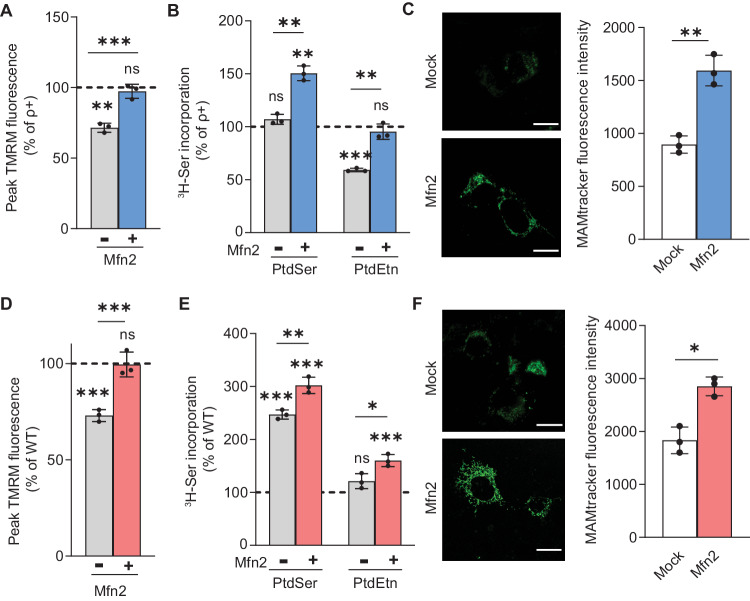
Increased MMP reverses deficiencies in ER-mitochondrial communication. **A** Quantitation of MMP in ρ^0^ mock-transfected or transfected with Mfn2 compared to that in ρ^+^ cells (dotted line) (*n* = 3). Note that ρ^0^ cells expressing *Mfn2* showed an increase in MMP to essentially normal levels. **B** Incorporation of ^3^H-Ser into ^3^H-PtdSer and ^3^H-PtdEtn in ρ^0^ cells expressing *Mfn2* compared to that in mock-transfected ρ^0^ cells (*n* = 3). Note that the decreased incorporation into ^3^H-PtdEtn in ρ^0^ cells was restored to essentially normal levels when *Mfn2* was expressed. **C** Representative confocal microscopy images of MAM (MAMtracker-Green, green) in ρ^0^ mock-transfected or transfected with *Mfn2*. Scale bars = 20 μm. Quantitation at right, as in Fig. 2E. Note increase in MAMtracker fluorescence intensity in ρ^0^ cells expressing *Mfn2*, in agreement with the phospholipid transfer assay shown in (**B**). **D** Quantitation of MMP in Δ-KSS cybrids mock-transfected or transfected with *Mfn2* compared to that in WT-KSS cells (dotted line) (*n* = 3). Note that Δ-KSS cells expressing *Mfn2* showed an increase in MMP to essentially normal levels, similar to what we observed in ρ^0^ cells. **E** Incorporation of ^3^H-Ser into ^3^H-PtdSer and 3H-PtdEtn in Δ-KSS cells expressing *Mfn2* compared to mock-transfected Δ-KSS cells (*n* = 3). Note the increase in ^3^H-Ser incorporation into ^3^H-PtdSer and ^3^H-PtdEtn in Δ-KSS when *Mfn2* was expressed. **F** Representative confocal microscopy images of MAM (MAMtracker-Green, green) in Δ-KSS cells mock-transfected or transfected with *Mfn2*. Scale bars = 20 μm. Quantitation at right, as in Fig. 2E. Note increase in MAMtracker fluorescence intensity in Δ-KSS cells expressing *Mfn2*, in agreement with the phospholipid transfer assay shown in (**E**).

Mutations in OxPhos complexes, and especially those associated with reduced MMP, appear to reduce ER-mitochondrial communication at the MAM. If so, increasing ER-mitochondrial communication in OxPhos-compromised cells should reverse MAM dysfunction, thereby confirming that pathogenic OxPhos mutations and reduced MMP indeed play a role in altering MAM functionality. To test this, we increased ER-mitochondrial connectivity by transfecting ρ^0^ cells and Δ-KSS cybrids (with MMP and reduced inter-organellar communication) with a plasmid encoding Mfn2 (Supplementary Fig. 2G), a protein that promotes ER-mitochondrial interactions [47, 48], and measured MMP and PL synthesis. ρ^0^ cells expressing *Mfn2* showed ~40% higher TMRM fluorescence intensity compared to mock-transfected ρ^0^ cells, to essentially normal levels (Fig. 7A). Concomitantly, we found that the decreased incorporation of ^3^H-Ser into ^3^H-PtdEtn in ρ^0^ cells (see also Fig. 1B) was restored to essentially normal levels when *Mfn2* was expressed (Fig. 7B). Importantly, MMP levels were also restored in Δ-KSS cybrids expressing *Mfn2* compared to mock-transfected Δ-KSS cells (Fig. 7D), with a corresponding increase in ER-mitochondria connectivity (PtdSer increased by 54% and PtdEtn by 40%; Fig. 7E). In line with this, we observed a corresponding increase in ER-mitochondrial apposition as measured by MAMtracker-Green in ρ^0^ cells and in Δ-KSS cybrids expressing *Mfn2* compared to their mock-transfected counterparts (Fig. 7C, F).

Taken together, these results imply that MAM functionality is indeed the most likely target (either direct or indirect) of the altered MMP associated with many mitochondrial disorders.

### Alterations in ER-mitochondrial connectivity contribute to cell death

**Fig. 8:**
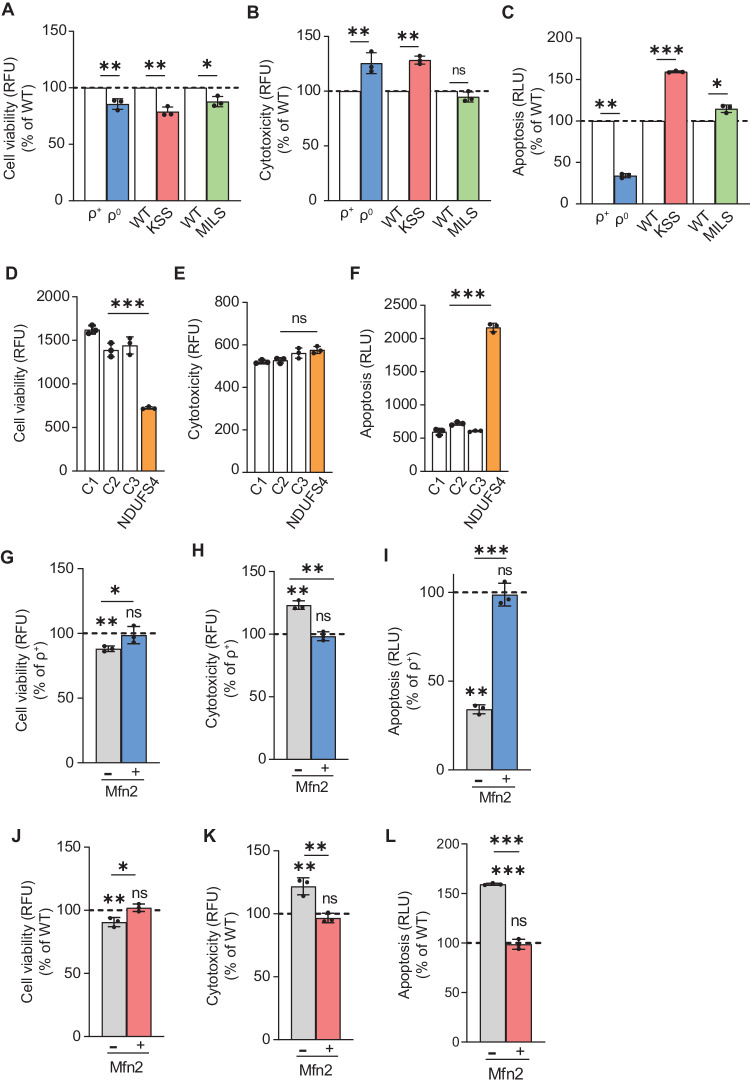
Alterations in MAM have consequences for cell survivability. **A** Quantitation of cell viability in ρ^0^, Δ-KSS and MILS cells relative to their WT counterparts (dotted line) (*n* = 3 independent experiments). Note that cell viability was reduced in all the mutant cells compared to their controls. **B** Quantitation of cytotoxicity in ρ^0^, Δ-KSS and MILS cells relative to their WT counterparts (dotted line) (*n* = 3 independent experiments). Note that cytotoxicity increased in ρ^0^ and Δ-KSS while no changes were observed in MILS cells compared to their controls. **C** Quantitation of apoptosis in ρ^0^, Δ-KSS and MILS cells relative to their WT counterparts (dotted line) (*n* = 3 independent experiments). Note that caspase activity was reduced in ρ0 cells while it was increased in Δ-KSS and MILS cybrids. **D** Quantitation of cell viability in NDUFS4 fibroblasts and control fibroblasts (C1, WT001; C2, KR003; C3, FC8) (*n* = 3 independent experiments). Note that cell viability was reduced in NDUFS4 fibroblast compared to the controls. **E** Quantitation of cytotoxicity in NDUFS4 fibroblasts and control fibroblasts. (*n* = 3 independent experiments). Note that cytotoxicity was unaltered in NDUFS4 fibroblasts compared to the controls. **F** Quantitation of apoptosis in NDUFS4 fibroblasts and control fibroblasts. Note that apoptosis significantly increased in NDUFS4 fibroblasts compared to the control fibroblasts. **G** Quantitation of cell viability in ρ^0^ mock-transfected or transfected with *Mfn2* compared to that in ρ^+^ cells (dotted line) (*n* = 3). Note that ρ^0^ cells expressing *Mfn2* showed an increase in cell viability to essentially normal levels. **H** Quantitation of cytotoxicity in ρ^0^ mock-transfected or transfected with *Mfn2* compared to that in ρ^+^ cells (dotted line) (*n* = 3). Note that ρ^0^ cells expressing Mfn2 restored the cytotoxicity to essentially normal levels. **I** Quantitation of apoptosis in ρ^0^ mock-transfected or transfected with *Mfn2* compared to that in ρ^+^ cells (dotted line) (*n* = 3). Note that ρ^0^ cells expressing *Mfn2* restored the apoptosis to essentially normal levels. **J** Quantitation of cell viability in Δ-KSS cybrids mock-transfected or transfected with *Mfn2* compared to that in WT-KSS cells (*n* = 3). Note that Δ-KSS expressing *Mfn2* showed an increase in cell viability to essentially normal levels. **K** Quantitation of cytotoxicity in Δ-KSS cybrids mock-transfected or transfected with *Mfn2* compared to that in WT-KSS cells (*n* = 3). Note that Δ-KSS expressing *Mfn2* restored the cytotoxicity to essentially normal levels. **L** Quantitation of apoptosis in Δ-KSS cybrids mock-transfected or transfected with *Mfn2* compared to that in WT-KSS cells (*n* = 3). Note that Δ-KSS cybrids expressing Mfn2 restored the apoptosis to essentially normal levels. RFU relative fluorescence units, RLU relative luminescence units.

MAM modulates multiple physiological events, including Ca^2+^ homeostasis, lipid synthesis and trafficking, and bioenergetics. Thus, it is not surprising that changes in these processes are common triggers of cell death, indicating a crucial role of MAM in regulating cell survival [49]. To understand the consequences of alterations in MAM connectivity, we measured cell viability (which reflects cell permeability), cytotoxicity (which reflects primary necrosis), and caspase activity (which reflects apoptosis) in the mitochondrial diseases studied here. Notably, we found a moderate decrease in cell viability in ρ^0^ cells, Δ-KSS, and MILS cybrids (by ~15%, ~20%, and ~12% respectively; Fig. 8A) compared to their WT counterparts. Similarly, cytotoxicity increased slightly in ρ^0^ cells and Δ-KSS cybrids (by ~25% and ~28% respectively; Fig. 8B), indicating the presence of necrotic cells, while in MILS cybrids no necrotic events were observed. Interestingly, caspase activity was reduced significantly in ρ^0^ cells (by ~60%), indicating that the classic caspase-dependent apoptosis pathway is inhibited in OxPhos-compromised cells. This observation aligns with a previous study that showed that ρ^0^ cells are resistant to apoptosis [50]. However, apoptosis was increased in Δ-KSS and MILS cybrids (by ~60% and ~15% respectively) compared to their WT counterparts (Fig. 8C), as reported previously [51, 52]. In addition, NDUFS4 fibroblasts showed a decrease in cell viability ( ~ 50%; Fig. 8D), no differences in cytotoxicity (Fig. 8E), and a significant increase in caspase activity (~4 fold; Fig. 8F) over control fibroblasts, suggesting that NDUFS4 fibroblasts undergo apoptosis.

Overall, these findings suggest, but of course do not prove, that alterations in ER-mitochondrial connectivity may be involved in cell survival in mitochondrial diseases, as cells with low ER-mitochondrial connectivity (ρ^0^ cells, KSS cybrids, and NDUFS4 fibroblasts; Figs. 1, 3 and 5) or high ER-mitochondrial connectivity (MILS cybrids; Fig. 4) presented signs of necrosis (as in ρ^0^ cells), apoptosis (as in MILS cybrids and NDUFS4 fibroblasts), or necrosis and apoptosis (as in KSS cybrids). In order to verify this assumption, we increased ER-mitochondrial connectivity by transfecting ρ^0^ cells and Δ-KSS cybrids with a plasmid encoding *Mfn2* (as described above), and measured cell viability, cytotoxicity, and caspase activity. Interestingly, we found that the decrease in cell viability and the increase in cytotoxicity in ρ^0^ cells and Δ-KSS cybrids was restored to essentially normal levels when *Mfn2* was expressed (Fig. 8G, H, J, K). Notably, apoptosis was also restored in ρ^0^ cells and Δ-KSS cybrids expressing *Mfn2* compared to mock-transfected Δ-KSS cells, with a corresponding increase of 65% in ρ^0^ cells and a decrease of 60% in Δ-KSS cybrids compared to their mock-transfected cells (Fig. 8I, L). Overall, these results indicate that alterations in ER-mitochondrial communication negatively impact cell survival in mitochondrial diseases.

## Discussion

For more than 30 years the field of mitochondrial disorders has been confronted by a “genotype-phenotype” problem. Since essentially all pathogenic mutations in mtDNA result in OxPhos deficiency, thereby causing mitochondrial disease, one might have expected that most, if not all, of these mutations would have a fundamentally similar clinical phenotype, but that is not the case [7]. For example, patients with mitochondrial encephalomyopathy, lactic acidosis, and stroke-like episodes (MELAS; mutation in tRNA^Leu(UUR)^) have the eponymous strokes, whereas patients with myoclonus epilepsy with ragged-red fibers (MERRF; mutations in tRNA^Lys^) have lipomas (but no strokes) [7]. Similarly, patients with KSS have heart conduction block not seen in other mtDNA disorders, and patients with mutations in tRNA^Ile^ have cardiopathy as its exclusive symptom [53]. Finally, patients with NARP/MILS (mutations in ATPase6) have Leigh syndrome at high mutant loads but retinitis pigmentosa at lower mutant levels [7]. Although all of these mutations compromise OxPhos, OxPhos deficiency alone almost certainly cannot explain the clinical variability seen in these disorders.

Recently, altered MAM function has been recognized as an important hallmark in a number of neurodegenerative diseases. Notably, in those disorders, altered MAM was associated with impaired bioenergetic output [17, 18, 54]. However, there is almost no information on the opposite possibility, namely, whether perturbed bioenergetics can affect MAM function [20, 55]. We now show that, in fact, cells with deficiencies in OxPhos show perturbed MAM functions, but surprisingly, not in a uniform way. Rather, each disorder studied by us apparently has a specific MAM “signature,” as measured by two well-recognized readouts, phospholipid synthesis/transport and cholesteryl ester synthesis [13]. Thus, like the genotype-phenotype problem, our results on MAM function do not fall into easily-defined categories, but nevertheless point to a new way of thinking about these devastating disorders.

For example, with respect to the dynamic synthesis of phospholipids, KSS cybrids showed an increase in PtdSer synthesis, but no change in PtdEtn synthesis, likely reflecting a severe deficiency in the ER-to-mitochondrial transport of PtdSer, similar to the reduction in the PtdEtn/PtdSer ratio that we observed in ρ^0^ cells (Figs. 2B and 3B). On the other hand, MILS cybrids showed an *increase* in the synthesis of both phospholipid species as well as in the conversion of PtdSer to PtdEtn (Fig. 4B), indicating the opposite outcome, namely increased ER-mitochondrial communication. In the NDUFS4 fibroblasts there was a decrease in the synthesis of both species (Fig. 5B). Notably, these results were consistent with our finding of parallel alterations in MAMtracker Green fluorescence in these cell types (Figs. 3C, 4C and 5C). Thus, in broad view, and based on these assays, ρ^0^ cells, KSS cybrids, and NDUFS4 fibroblasts had less ER-mitochondrial communication than did their WT counterparts, whereas MILS cybrids had more.

The PL synthesis data, however, tell only half the story. Using cholesteryl ester synthesis and lipid droplet formation as another readout for MAM function, we observed a decrease in CE synthesis in ρ^0^ cells and in MILS cybrids (Figs. 2B and 4D), and instead of seeing a parallel decrease in lipid droplets, we found an increase, and these LDs contained predominantly triglycerides (TGAs), not CEs (Figs. 2E and 4G). On the other hand, KSS cybrids and NDUFS4 cells showed an increase in CEs (Figs. 3D and 5D), but only the KSS cells showed a corresponding increase in LDs (Figs. 3E, F and 5E, F). The reason for this pattern is unclear, but we note that all of the cell types analyzed showed an increase in lipid deposition of one type or another (i.e., CEs or TGAs or both), suggesting a robust activation of lipid remodeling pathways in mitochondrial diseases, presumably to promote membrane repair [56, 57].

While there was a direct correlation between the alteration in PtdEtn synthesis and ER-mitochondrial connectivity in these cells, the changes in overall lipid metabolism - in CEs, TGAs, and LDs - were independent of ER-mitochondrial apposition. In fact, this lack of correlation may shed light on the way MAM functions: PtdEtn synthesis requires trafficking of PtdSer from the ER (at MAM), where PtdSer is synthesized [37], to mitochondria, where it is converted to PtdEtn [38] (Fig. 1A). Thus, phospholipid synthesis/trafficking may be considered to be a “vertical” function, as PtdSer is transferred from ER-MAM to mitochondria and re-transported back to the ER-MAM [38]. In contrast, CE and TGA synthesis may be considered to be “horizontal” functions, as both activities reside exclusively in the ER-MAM (by ACAT1 and DGAT2, respectively) [27, 43], with mitochondria playing essentially no metabolic role. This “horizontal/vertical” hypothesis of MAM functionality [13, 58] remains under study.

Our analyses point to a number of conclusions regarding the relationship between mitochondrial diseases and ER-mitochondrial communication. First, altered MAM function in NDUFS4 cells implies that the origin of the mutation (i.e., mtDNA vs nDNA) plays little, if any, role in this phenomenon. Second, the fact that the MAM phenotype of reduced connectivity in KSS cybrids (containing a partial deletion of mtDNA) mimics that in ρ^0^ cells (lacking mtDNA) implies that mtDNA and/or mitochondrial nucleoids per se is not involved in the regulation of ER-mitochondrial connectivity. Finally, our finding in the MILS cybrids of a result opposite to that in KSS and ρ^0^ cells, namely increased communication, points to a potential mechanism that might explain how altered OxPhos can affect MAM behavior. Specifically, a major difference between most pathogenic mtDNA mutations and those causing MILS is that the former typically compromise electron transport and reduce mitochondrial membrane potential, whereas the latter do not compromise R.C. function and not only do not decrease MMP, but in fact increase it [59].

A role for reduced MMP in triggering reduced ER-mitochondrial connectivity is supported by four lines of evidence. First, MMP was reduced in WT ρ^+^ cells when complexes I, III, and IV (all of which pump protons, thereby contributing to the MMP) were inhibited pharmacologically, but not when complex II (which does not contribute to the MMP, as it transfers electrons but does not pump protons) was inhibited. (As an aside, the lack of an effect on complex II implies that the electron transport chain per se does not play a role in regulating MAM function.) Second, addition of uncouplers (that dissipate the MMP) to WT ρ^+^ cells with baseline levels of MMP and ER-mitochondrial communication resulted in a significant reduction in both parameters. Third, the same result was obtained in MILS cybrids having above-normal levels of both MMP and inter-organellar communication (Fig. 6H, J, K). This latter result is remarkable, as it implies a correlation between MMP and ER-mitochondrial communication (correlation, of course, does not imply causation). Fourth, increasing ER-mitochondrial connectivity via Mfn2 overexpression (which promotes tethering at the ER-mitochondria interface) [47] reversed the loss of MMP in ρ^0^ cells and in KSS cybrids to essentially normal levels (Fig. 7A–D), supporting the idea that it is indeed ER-mitochondrial connectivity that is impaired upon loss of MMP.

The reason for a relationship between MMP and ER-mitochondrial communication is unknown. While a number of possibilities present themselves, all fundamentally based on a postulated modification of [membrane-associated] proteins by altered MMP [60–64], we lean towards a more biophysical explanation in which alteration in repulsive or attractive forces between the mitochondrial outer membrane (MOM) and the ER at mitochondria-ER contact sites (MERCs [65]; might govern their connectivity [66]. Specifically, it has long been known that a change in transmembrane potential across a lipid bilayer (due to fluctuations in the charge distribution within the bilayer leaflets) changes the lipid composition of the membrane [66, 67] and the degree of attraction between two apposed membranes [68]; the remodeling of mitochondrial membranes by MMP would presumably be no different than that of other cellular membranes [69]. Thus, when MMP is altered - either reduced (as in KSS) or increased (as in MILS) - it is conceivable that there is a remodeling of the membrane lipids at the MOM-MAM interface (as documented here) such that the apposition between the two membranes is altered correspondingly (either repelled [as in KSS] or attracted [as in MILS]).

Lastly, alterations in ER-mitochondrial connectivity affected cell survival in each disorder (Fig. 8A–F). Cells in which MAM was reduced (as in ρ^0^, Δ-KSS, and NDUFS4 fibroblasts) or overexpressed (as in MILS) showed a necrotic and/or apoptotic profile which was reversed to essentially normal levels following an increase in ER-mitochondrial communication via Mfn2 overexpression (Fig. 8G–L). Although the relationship between ER-mitochondrial connectivity and cell death is poorly understood, a dysregulation in lipid metabolism by MAM has been associated with cell death [70, 71]. In line with this, a recent proposal has emerged suggesting that changes in the composition of the lipid raft may be linked to the pathology of KSS patients, as several apoptotic pathways are mediated by lipid rafts [72]. Specifically, alterations in the levels of 5-methyltetrahydrofolate (5-MTHF), free sialic acid, sphingomyelin, and tau protein suggest the involvement of lipid rafts in mitochondrial dysfunction, ultimately leading to cell death and contributing to the development of KSS [73, 74]. Thus, we can postulate that alterations in ER-mitochondrial connectivity can induce pathological changes in the lipid composition at the MAM, a lipid raft [13], inducing lipid dyshomeostasis, which ultimately leads to cell death.

Our observation of the effect of OxPhos deficiency on ER-mitochondrial communication is mirrored by the finding that alterations in ER-mitochondrial communication can cause alterations in OxPhos efficiency [17, 18]. This reciprocal behavior implies that from a functional standpoint, ER-mitochondrial communication is “bidirectional,” raising the issue of cause-and-effect (i.e., altered OxPhos and MMP affect MAM; altered MAM affects OxPhos and MMP). If true, this conclusion implies that more than one mechanism modulates the “OxPhos-MAM” axis. It also implies that it may be possible to improve OxPhos in mitochondrial disease cells (especially those with heteroplasmic mtDNA mutations) by renormalizing ER-mitochondrial connectivity.

Finally, as with most early-stage discoveries, many of the findings presented here are descriptive, but we note that the findings regarding the role of MMP in mediating MAM dysfunction already point to the beginnings of a mechanistic understanding of this phenomenon. Experiments to explore this possibility are underway.

### Supplementary information

Supplementary Fig. 1–5

Supplementary Figure legend

western Blots

## Data Availability

Data that form the basis of this report are available upon request.
