# Supplementary material for: Aberrant ER-mitochondria communication is a common pathomechanism in mitochondrial disease
Source: Cell Death Dis. 2024 Jun 10;15(6):405. doi: 10.1038/s41419-024-06781-9 (PMC11164949; doi:10.1038/s41419-024-06781-9)
Supplement: Supplementary file 1 — Supplementary Fig. 1–5 [file 41419_2024_6781_MOESM1_ESM.pdf]

EV 1

A

Cell line	Full code name	Mutation	Reference
ρ+	143B	None	King, Attardi 1989
ρ0	143B206	mtDNA depletion	King, Attardi 1989
WT-KSS	FLP6a39.2	None	Santra et al., 2004
Δ-KSS	FLP6a39.32	Deletion of mtDNA	Santra et al., 2004
WT-MILS	JCP 213	None	Palloti et al., 2004
Δ-MILS	JCP261	ATPase 6	Palloti et al., 2004

Fibroblast	Code	Mutation	Age	Sex
FC8	Control	None	35	Female
WT001	Control	None	3	Female
KR003	Control	None	3	Female
Δ-NDUFS4	NM16-019	355G>A (p. Asp119His)	3	Female

B

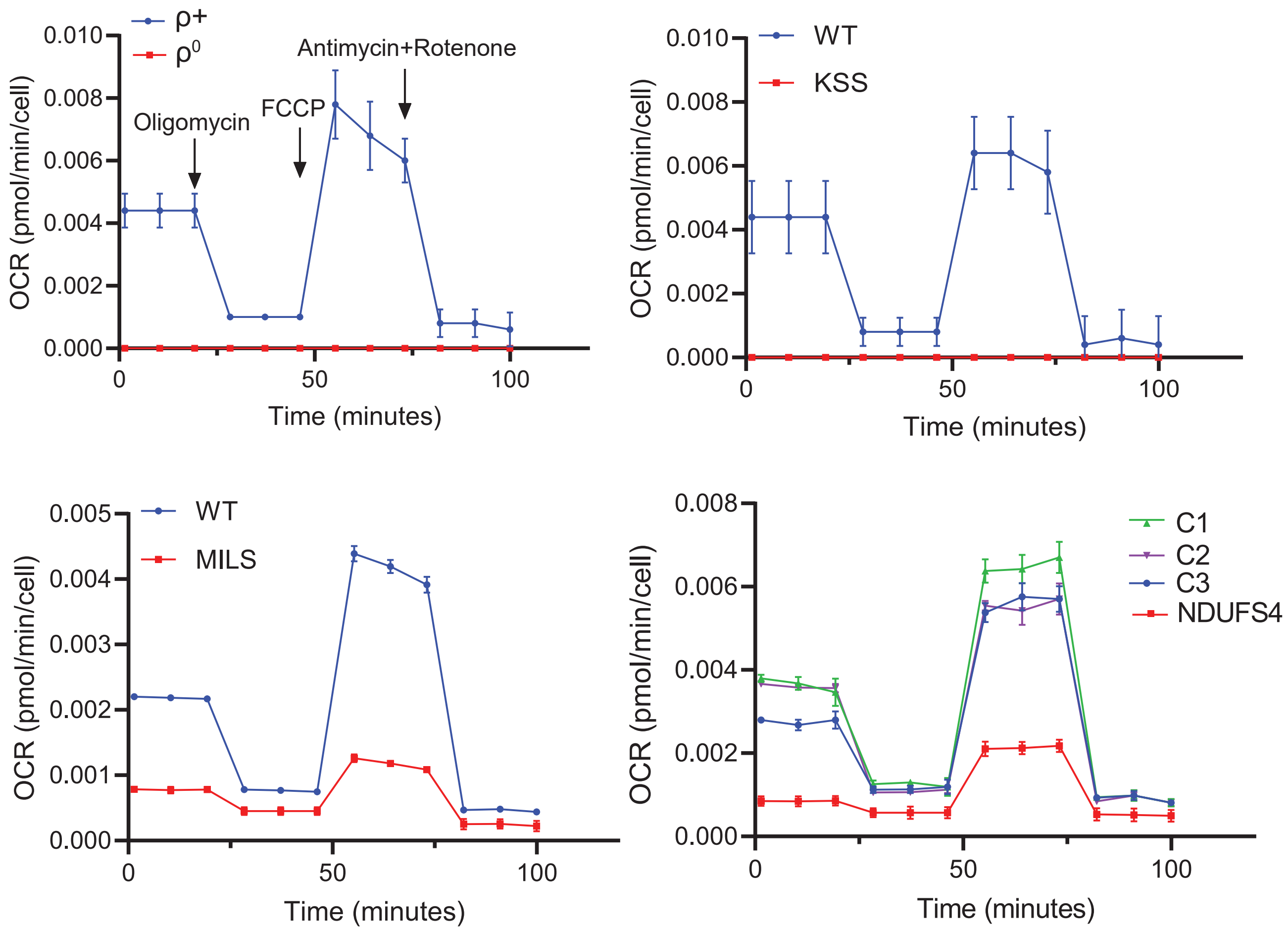

EV 2

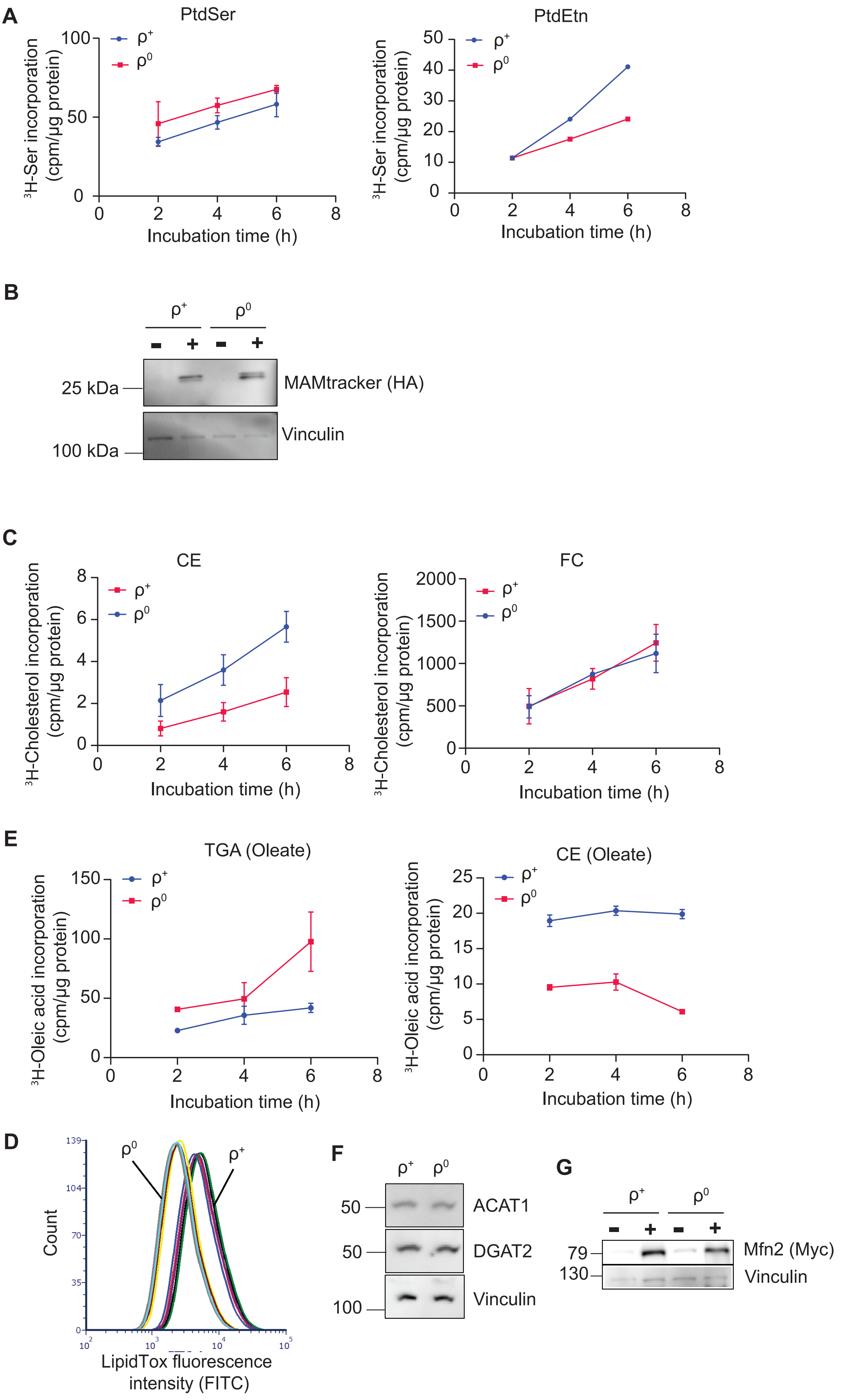
A
PtdSer
ρ+
ρ0
3H-Ser incorporation (cpm/µg protein)
100
50
0
0 2 4 6 8
Incubation time (h)
PtdEtn
ρ+
ρ0
3H-Ser incorporation (cpm/µg protein)
50
40
30
20
10
0
0 2 4 6 8
Incubation time (h)
B
ρ+
ρ0
- + - +
25 kDa
MAMtracker (HA)
Vinculin
100 kDa
C
CE
ρ+
ρ0
3H-Cholesterol incorporation (cpm/µg protein)
8
6
4
2
0
0 2 4 6 8
Incubation time (h)
FC
ρ+
ρ0
3H-Cholesterol incorporation (cpm/µg protein)
2000
1500
1000
500
0
0 2 4 6 8
Incubation time (h)
E
TGA (Oleate)
ρ+
ρ0
3H-Oleic acid incorporation (cpm/µg protein)
150
100
50
0
0 2 4 6 8
Incubation time (h)
CE (Oleate)
ρ+
ρ0
3H-Oleic acid incorporation (cpm/µg protein)
25
20
15
10
5
0
0 2 4 6 8
Incubation time (h)
D
ρ0
ρ+
Count
139
104
70
35
0
10^2 10^3 10^4 10^5
LipidTox fluorescence intensity (FITC)
F
ρ+ ρ0
50
ACAT1
50
DGAT2
100
Vinculin
G
ρ+
ρ0
- + - +
79
Mfn2 (Myc)
130
Vinculin

Supplementary Fig. 3

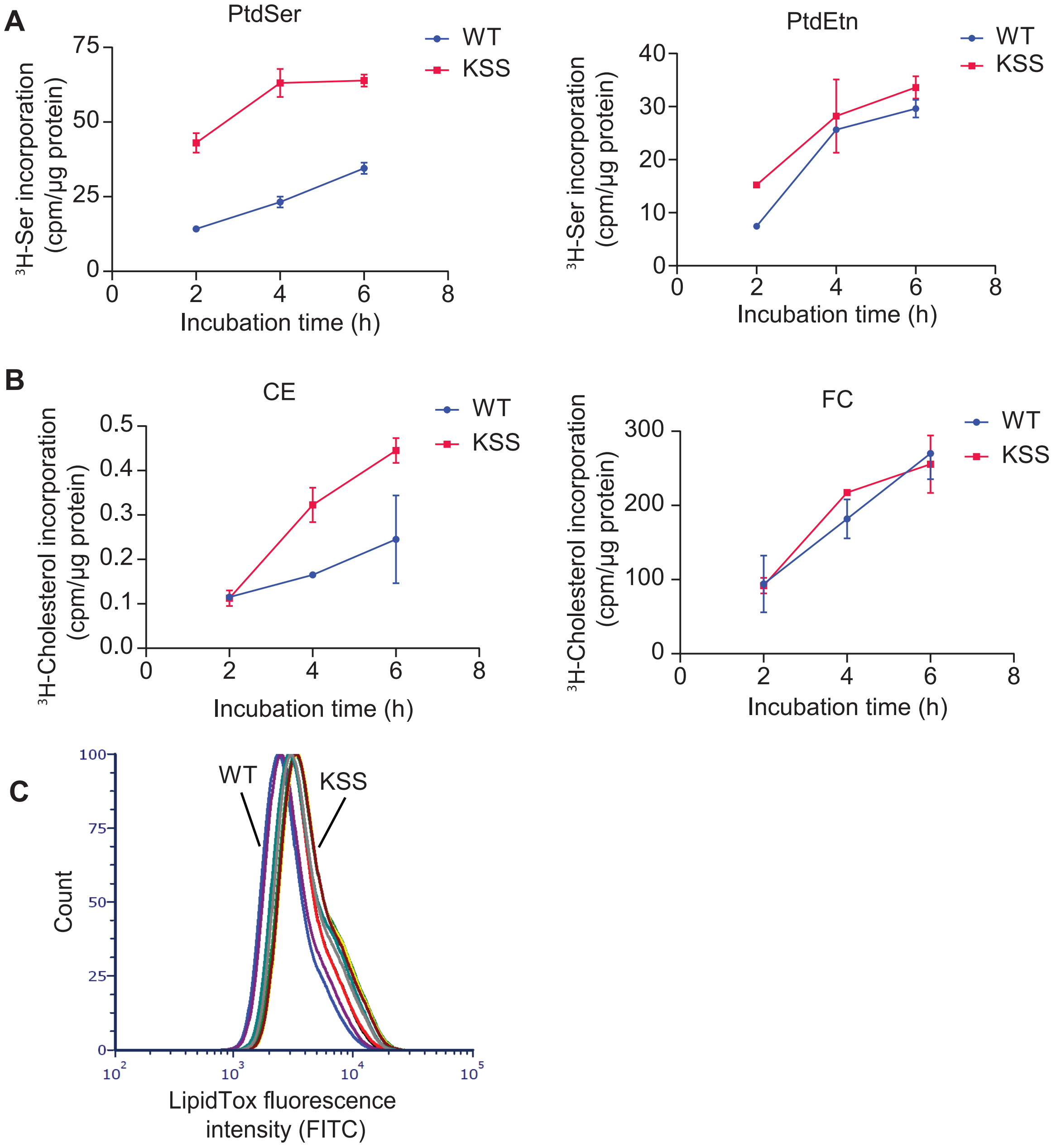

Supplementary Fig. 4

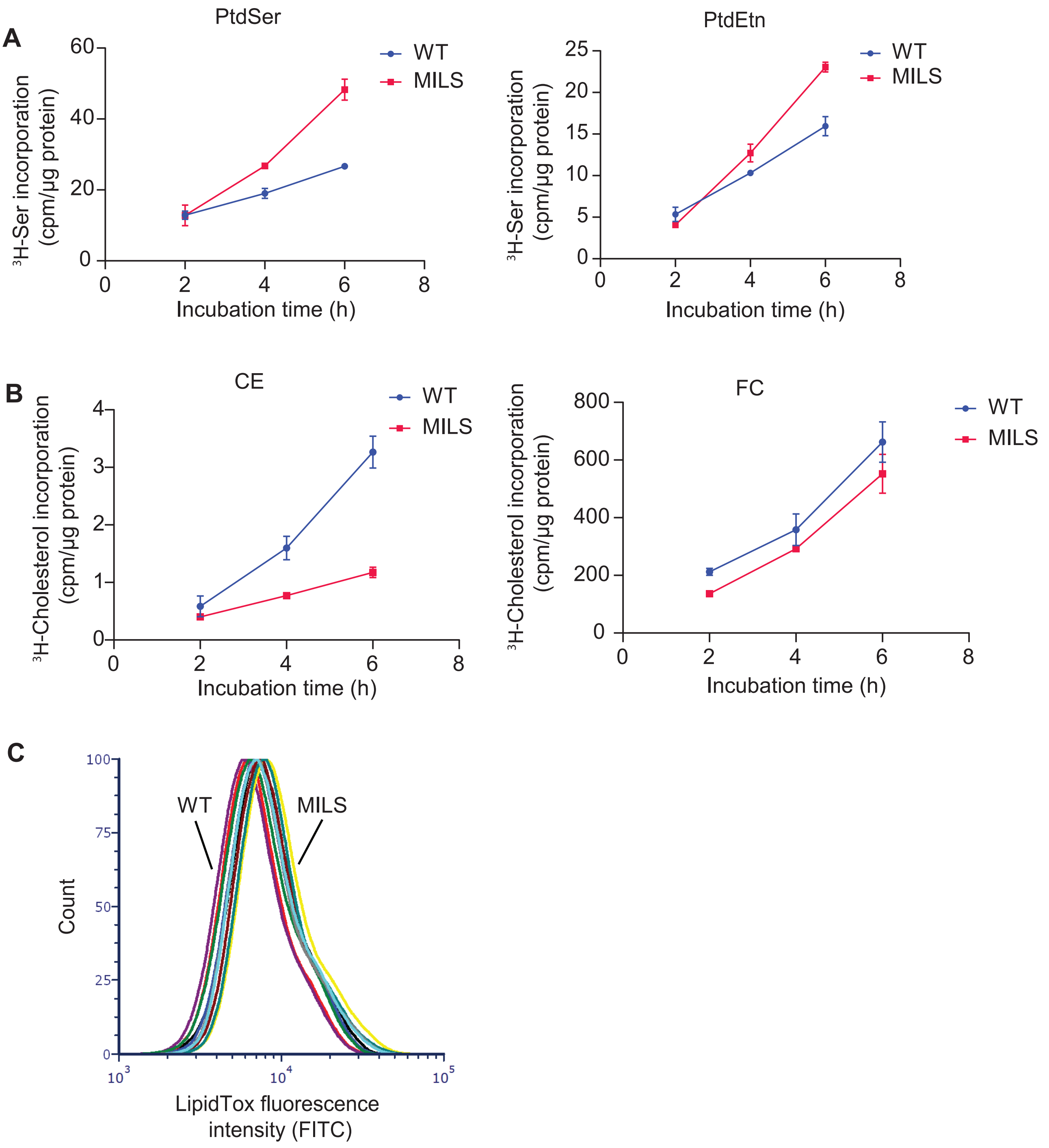

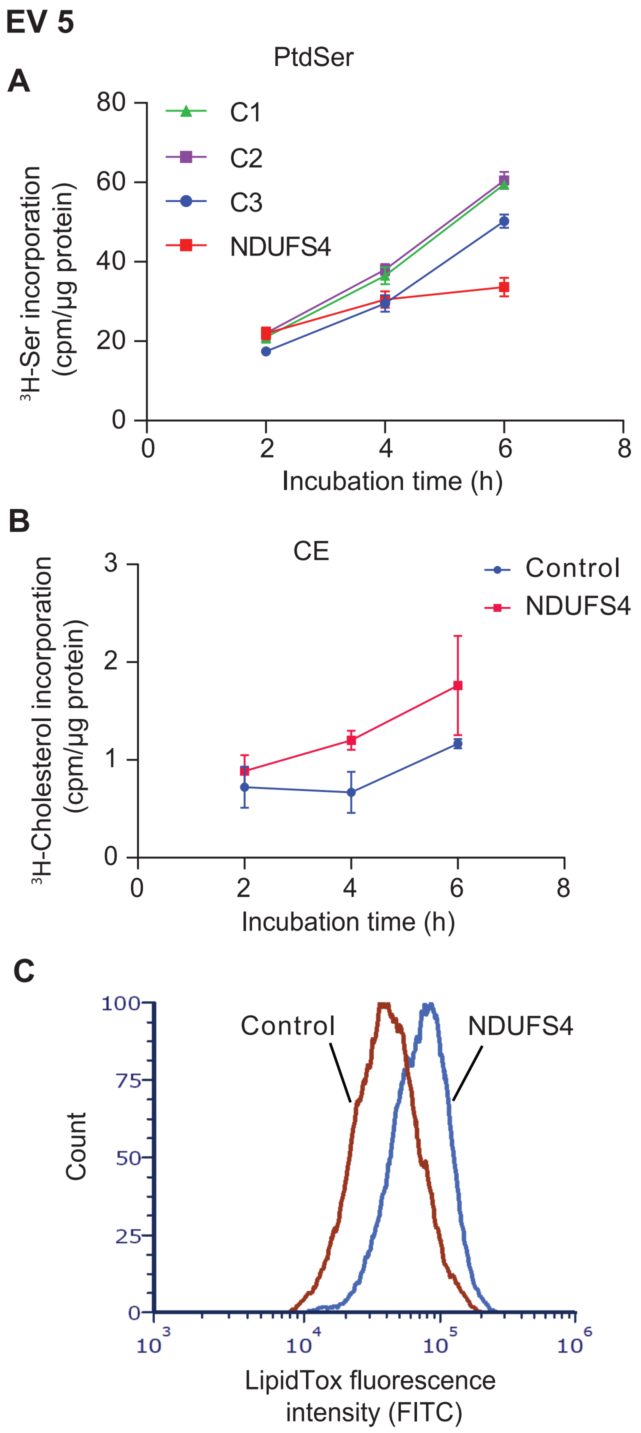
EV 5
A
PtdSer
80
60
40
20
0
C1
C2
C3
NDUFS4
3H-Ser incorporation (cpm/µg protein)
0 2 4 6 8
Incubation time (h)
PtdEtn
15
10
5
0
C1
C2
C3
NDUFS4
3H-Ser incorporation (cpm/µg protein)
0 2 4 6 8
Incubation time (h)
B
CE
3
2
1
0
Control
NDUFS4
3H-Cholesterol incorporation (cpm/µg protein)
0 2 4 6 8
Incubation time (h)
FC
1000
800
600
400
Control
NDUFS4
3H-Cholesterol incorporation (cpm/µg protein)
0 2 4 6 8
Incubation time (h)
C
100
75
50
25
0
Count
Control
NDUFS4
10^3 10^4 10^5 10^6
LipidTox fluorescence intensity (FITC)
