# Supplementary material for: Aberrant ER-mitochondria communication is a common pathomechanism in mitochondrial disease
Source: Cell Death Dis. 2024 Jun 10;15(6):405. doi: 10.1038/s41419-024-06781-9 (PMC11164949; doi:10.1038/s41419-024-06781-9)
Supplement: Supplementary file 2 — Supplementary Figure legend [file 41419_2024_6781_MOESM2_ESM.pdf]

Supplementary Figure 1. Mitochondrial respiration is decreased in OxPhos compromised cells. (A) Cell lines used in this study. **(B)** Representative time course of OCR measurements in the indicated pairs of cells using a Seahorse extracellular flux analyzer (n=3). Note that ρ^0 and Δ-KSS cells have essentially no respiratory chain function.

Supplementary Figure 2. Analysis of MAM function in ρ^0 cells. **(A)** Representative kinetics of the incorporation of ^{3}H-Ser into ^{3}H-PtdSer and ^{3}H-PtdEtn for the indicated times in ρ^+ and ρ^0 cells (n=4). **(B)** Western blot confirming the expression of MAMtracker-Green in the transfected cells, detected with anti-HA. **(C)** Representative kinetics of ^{3}H-cholesterol (free cholesterol; FC) and ^{3}H-CE (cholesteryl ester; CE) synthesis for the indicated times (n=4) in ρ^+ and ρ^0 cells. **(D)** Representative plot of LD formation following staining with LipidTox Green and quantitation by flow cytometry (n=3). **(E)** Representative kinetics of the conversion of ^{3}H-oleic acid to ^{3}H-TGA and ^{3}H-CE for the indicated times in ρ^+ and ρ^0 cells (n=4). **(F)** Representative Western blot of LD-relevant proteins (ACAT1 and DGAT2) compared to vinculin. **(G)** Western blot confirming the expression of Mfn2-Myc in the transfected cells, detected by anti-Myc.

Supplementary Figure 3. Analysis of MAM function in KSS cybrids. **(A)** Representative kinetics of the incorporation of ^{3}H-Ser into ^{3}H-PtdSer and ^{3}H-PtdEtn in WT-KSS and Δ-KSS cybrids for the indicated times (n=4). **(B)** Representative kinetics of ^{3}H-cholesterol and ^{3}H-CE synthesis for the indicated times (n=4) in WT-KSS and Δ-KSS cybrids. **(C)** Representative plot of LD formation following staining with LipidTox Green and quantitation by flow cytometry (n=3).

Supplementary Figure 4. Analysis of MAM function in MILS cybrids. **(A)** Representative kinetics of the incorporation of ^{3}H-Ser into ^{3}H-PtdSer and ^{3}H-PtdEtn in WT-MILS and mut-MILS cybrids for the indicated times (n=4). **(B)** Representative kinetics of ^{3}H-cholesterol and ^{3}H-CE synthesis for the indicated times (n=4) in WT-MILS and mut-MILS cybrids. **(B)** Representative plot of LD formation following staining with LipidTox Green and quantitation by flow cytometry (n=3) in WT-MILS and mut-MILS cybrids.

Supplementary Figure 5. Analysis of MAM function in NDUFS4 fibroblasts. **(A)** Representative kinetics of the incorporation of ^{3}H-Ser into ^{3}H-PtdSer and ^{3}H-PtdEtn in controls and NDUFS4 fibroblasts for the indicated times (n=4). **(B)** Representative kinetics of ^{3}H-cholesterol and ^{3}H-CE synthesis for the indicated times (n=4) in control and NDUFS4 fibroblasts. **(C)** Representative plot of LD formation following staining with LipidTox Green and quantitation by flow cytometry (n=3) in control and NDUFS4 fibroblasts.
