# Supplementary material for: Aberrant ER-mitochondria communication is a common pathomechanism in mitochondrial disease
Source: Cell Death Dis. 2024 Jun 10;15(6):405. doi: 10.1038/s41419-024-06781-9 (PMC11164949; doi:10.1038/s41419-024-06781-9)
Supplement: Supplementary file 3 — western Blots [file 41419_2024_6781_MOESM3_ESM.pptx]

## Slide 1
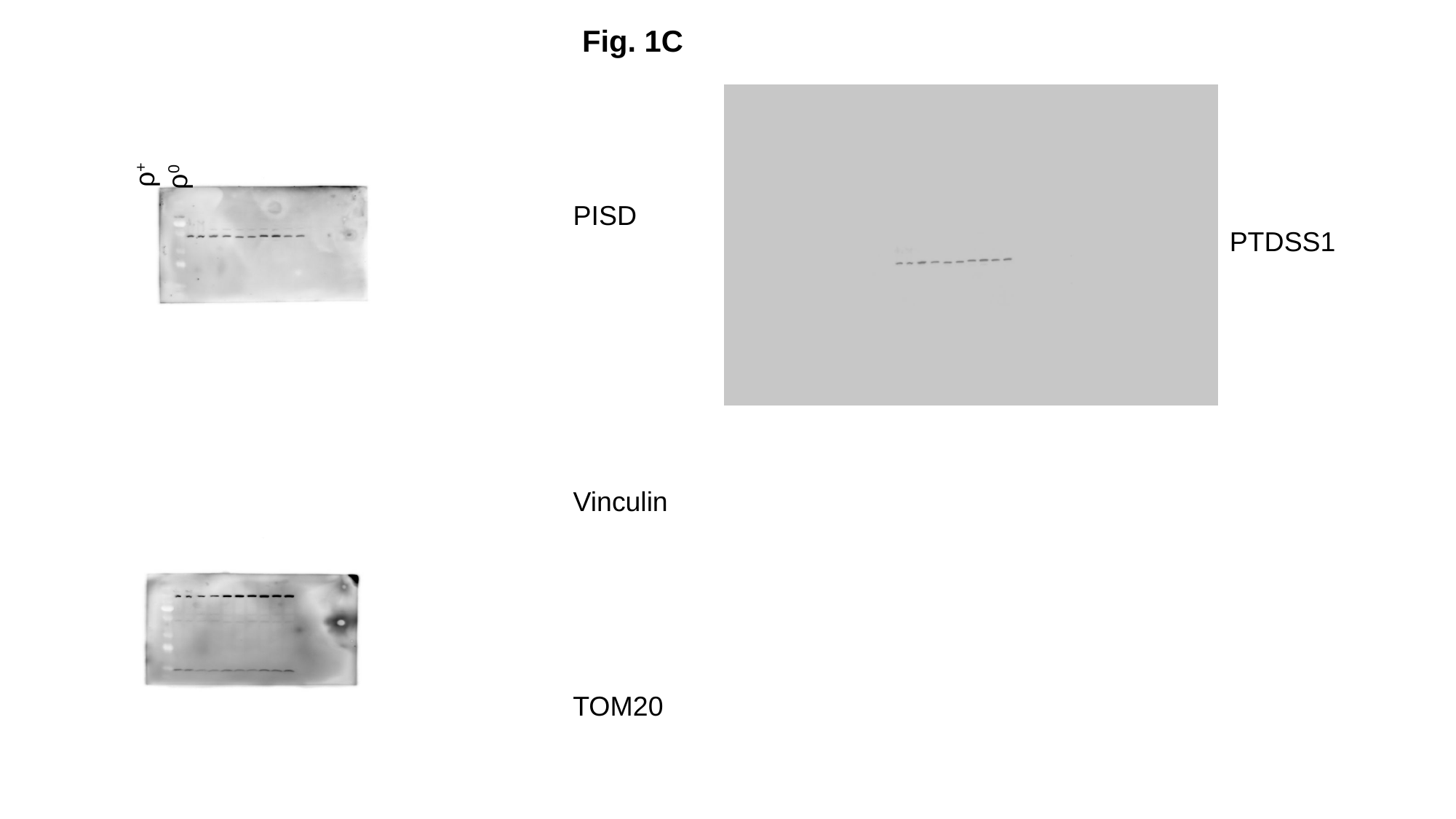

Fig. 1C
ρ0
ρ+
PISD
PTDSS1
Vinculin
TOM20

## Slide 2
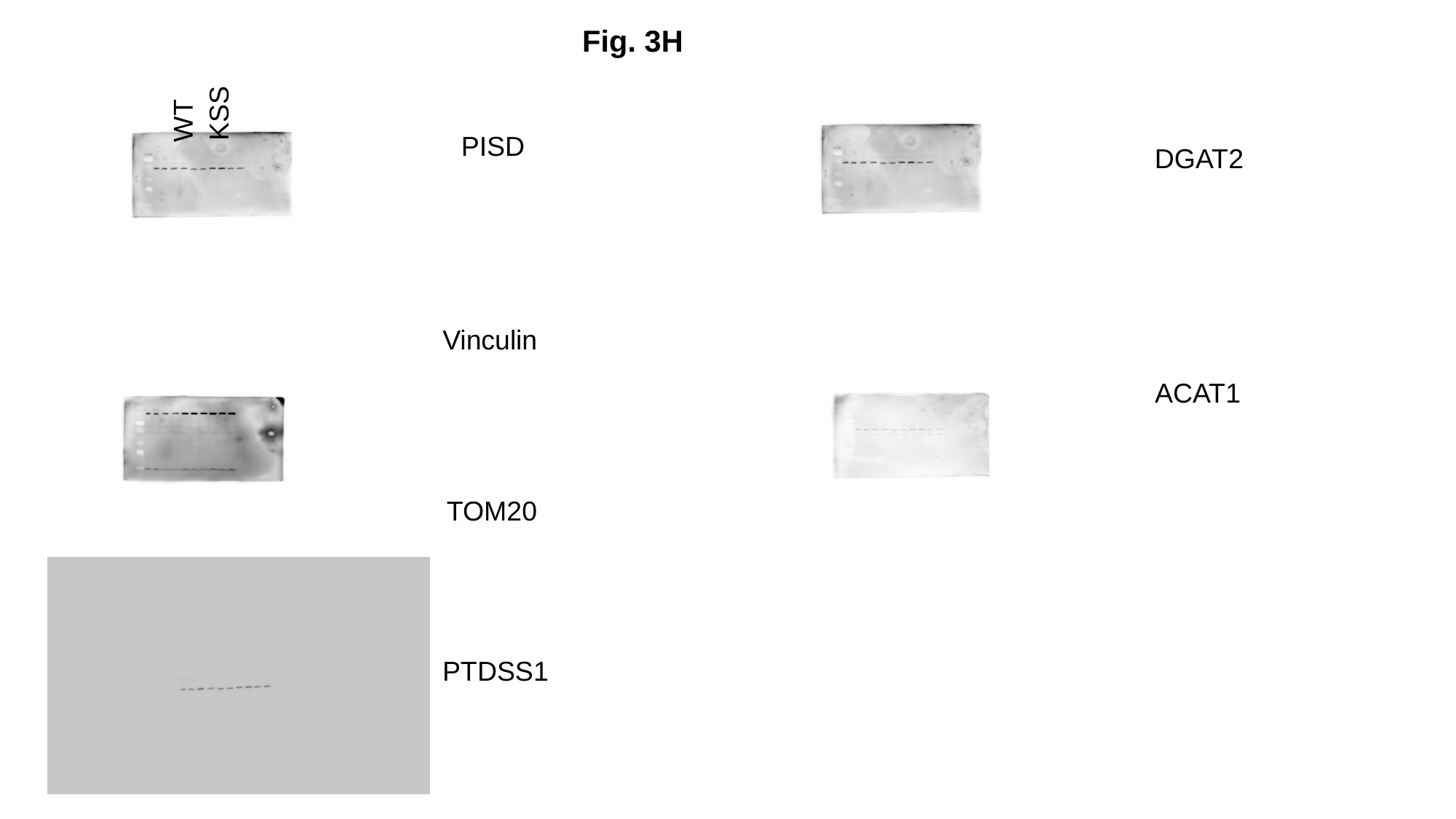

Fig. 3H
KSS
WT
PISD
DGAT2
Vinculin
ACAT1
TOM20
PTDSS1

## Slide 3
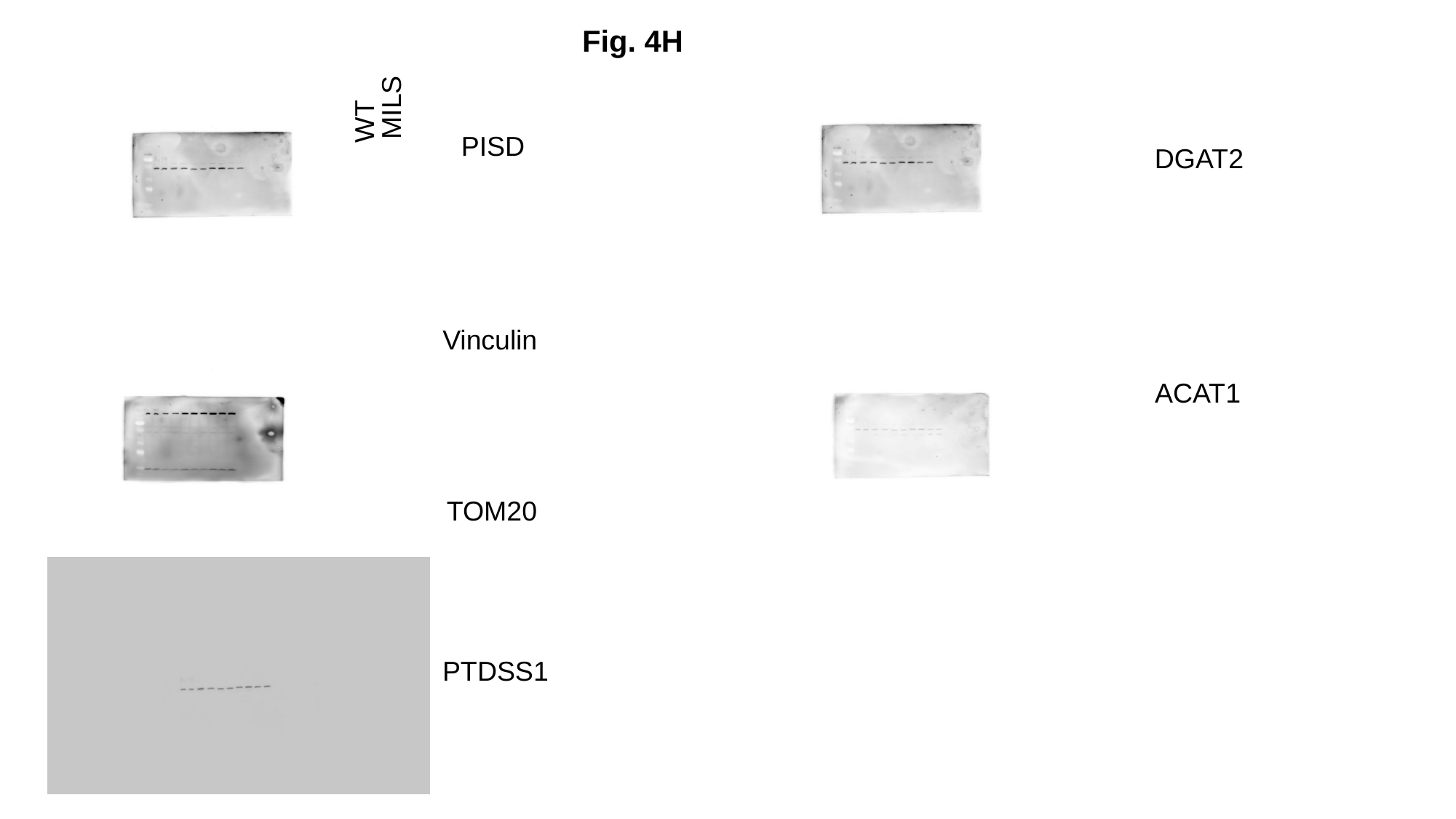

Fig. 4H
MILS
WT
PISD
DGAT2
Vinculin
ACAT1
TOM20
PTDSS1

## Slide 4
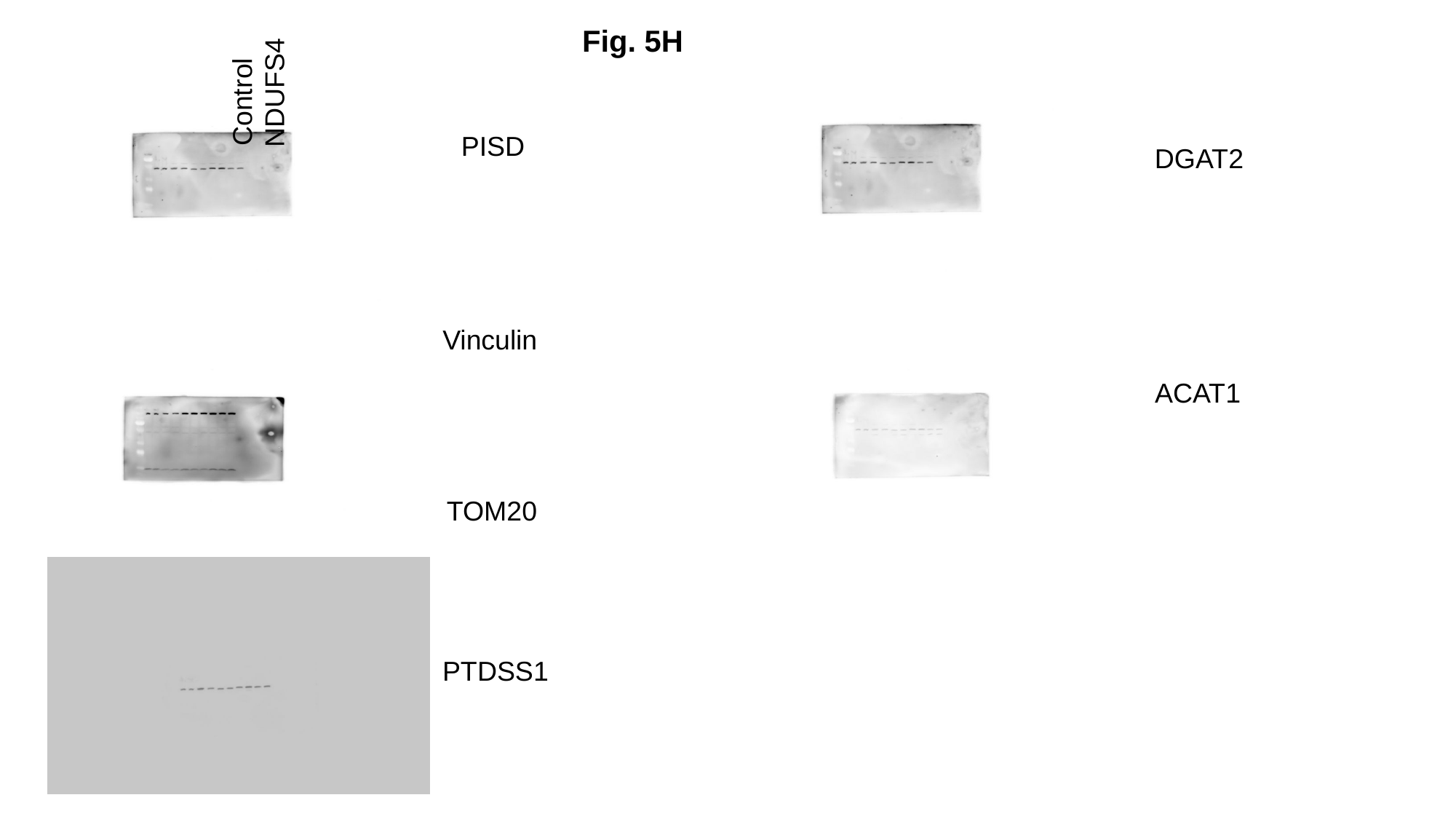

Fig. 5H
Control
NDUFS4
PISD
DGAT2
Vinculin
ACAT1
TOM20
PTDSS1

## Slide 5
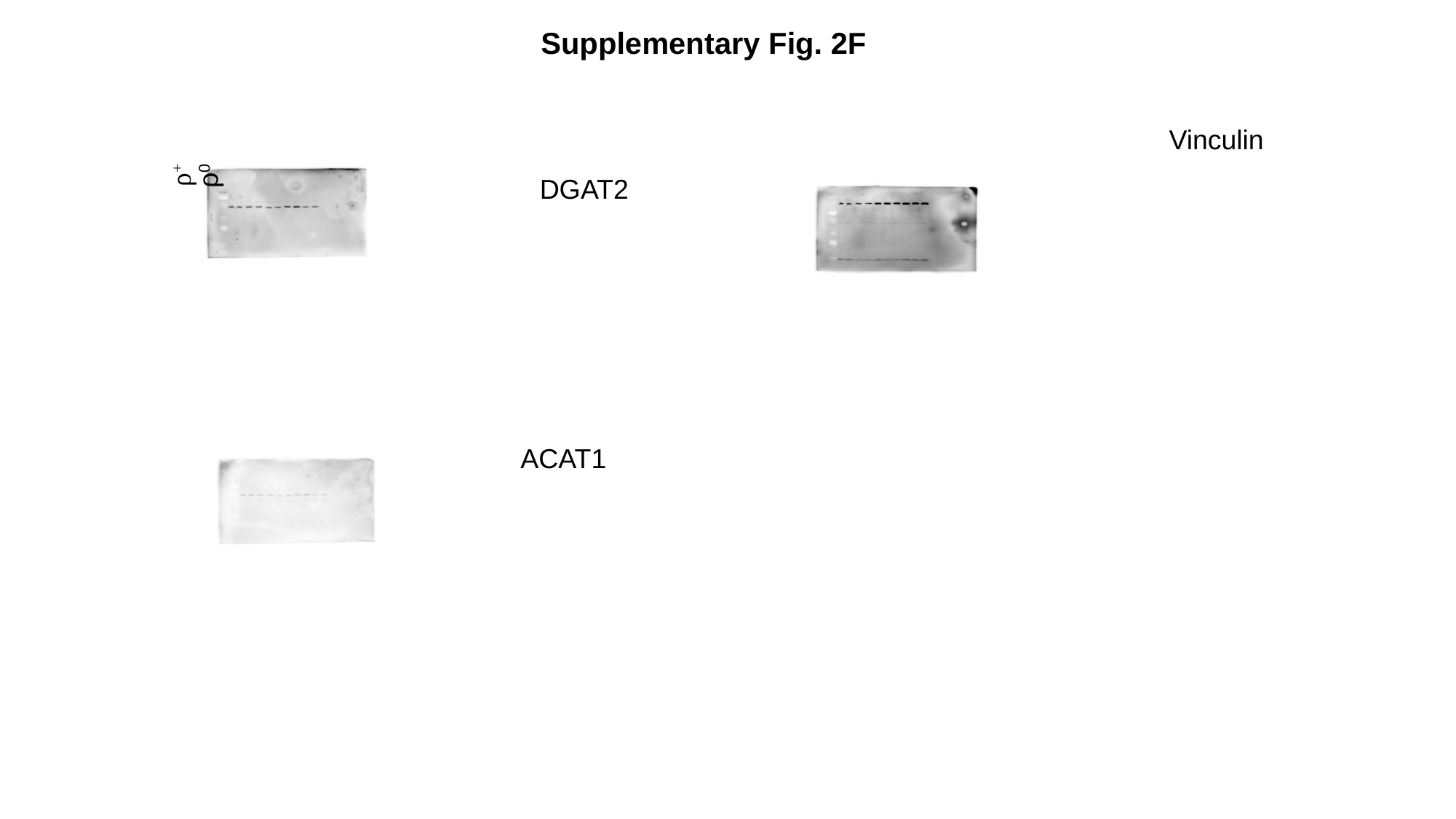

Supplementary Fig. 2F
Vinculin
ρ0
ρ+
DGAT2
ACAT1

## Slide 6
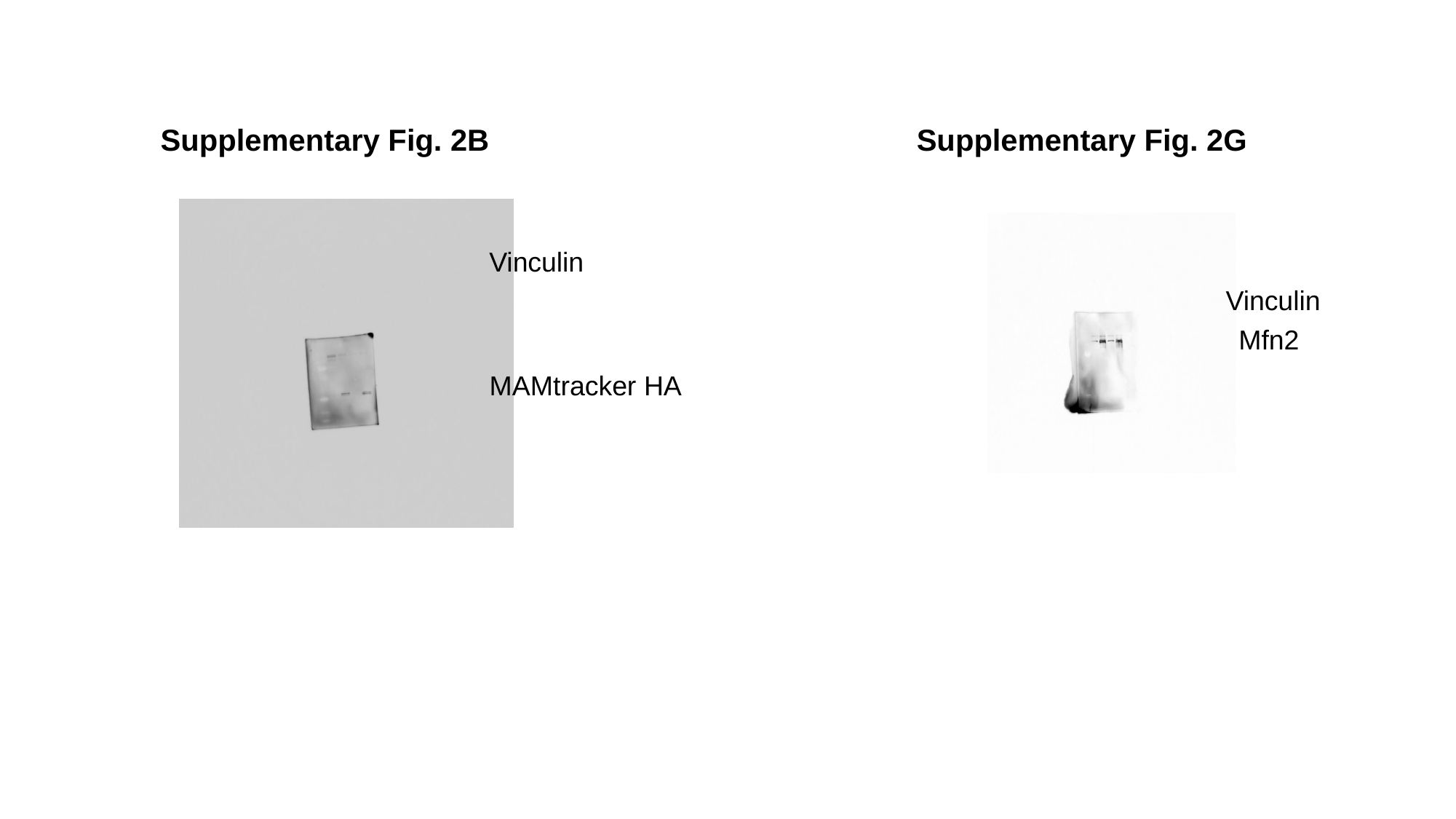

Supplementary Fig. 2G
Supplementary Fig. 2B
Vinculin
Vinculin
Mfn2
MAMtracker HA
